# Avian reovirus p17 and σA act cooperatively to downregulate Akt by suppressing mTORC2 and CDK2/cyclin A2 and upregulating proteasome PSMB6

**DOI:** 10.1038/s41598-017-05510-x

**Published:** 2017-07-12

**Authors:** Wei-Ru Huang, Pei-I Chi, Hung-Chuan Chiu, Jue-Liang Hsu, Brent L. Nielsen, Tsai-Ling Liao, Hung-Jen Liu

**Affiliations:** 1 Institute of Molecular Biology, National Chung Hsing University, Taichung, 402 Taiwan; 2 Agricultural Biotechnology Center, National Chung Hsing University, Taichung, 402 Taiwan; 3 Rong Hsing Research Center for Translational Medicine, National Chung Hsing University, Taichung, 402 Taiwan; 4 Department of Biological Science and Technology, National Pingtung University of Science and Technology, Pingtung, 912 Taiwan; 5 Department of Microbiology and Molecular Biology, Brigham Young University, Provo, Utah USA; 6 Department of Medical Research, Taichung Veterans General Hospital, Taichung, 407 Taiwan

## Abstract

Although we have shown that avian reovirus (ARV) p17-mediated inhibition of Akt leads to induction of autophagy, the precise mechanisms remain largely unknown. This study has identified a specific mechanism by which ARV coordinately regulates the degradation of ribosomal proteins by p17-mediated activation of E3 ligase MDM2 that targets ribosomal proteins and by σA-mediated upregulation of proteasome PSMB6. In addition to downregulating ribosomal proteins, p17 reduces mTORC2 assembly and disrupts mTORC2-robosome association, both of which inactivate mTORC2 leading to inhibition of Akt phosphorylation at S473. Furthermore, we discovered that p17 binds to and inhibits the CDK2/cyclin A2 complex, further inhibiting phosphorylation of Akt S473. The negative effect of p17 on mTORC2 assembly and Akt phosphorylation at S473 is reversed in cells treated with insulin or overexpression of CDK2. The carboxyl terminus of p17 is necessary for interaction with CDK2 and for induction of autophagy. Furthermore, p17-mediated upregulation of LC3-II could be partially reversed by overexpression of CDK2. The present study provides mechanistic insights into cooperation between p17 and σA proteins of ARV to negatively regulate Akt by downregulating complexes of mTORC2 and CDK2/cyclin A2 and upregulating PSMB6, which together induces autophagy and cell cycle arrest and benefits virus replication.

## Introduction

The most predominant proteasome in mammals is the 26S proteasome, which consists of one 20S subunit, the catalytic part of the proteasome, and two 19S regulatory cap subunits^1–3^. The 19S regulatory subunit is responsible for stimulating the 20S subunit to degrade proteins. The 19S regulatory particle recognizes the polyubiquitin tag on the targeted substrates and unfolds the substrate to allow entry into the proteolytic chamber of the 20S core particle, which possesses the catalytic sites involved in proteolysis^4^.

Akt protein kinase plays key roles in cell proliferation, survival and metabolism. It has been established that Akt activity is regulated via phosphorylation at T308 and S473 by PDK1 and the mammalian target of rapamycin complex 2 (mTORC2)-ribosome, respectively^5, 6^. It has been demonstrated that active mTORC2 is physically associated with the ribosome^7^. More recently, the study by Liu *et al*. has advanced our understanding of the mechanism underlying activation of Akt^6^. This study demonstrated that phosphorylation of Akt at S477 and T479 by the CDK2/cyclin A complex enhances Akt activation by functionally compensating for Akt S473 phosphorylation. Furthermore, Akt phosphorylates Beclin 1 enhancing its interactions with 14-3-3 and vimentin, thereby inhibiting autophagy^8^. These observations have implications for understanding the regulation of Akt signaling and intermediate filament proteins in autophagy and cancer. It is well-known that p53, phosphatase and tensin homolog deleted on chromosome 10 (PTEN), and retinoblastoma (Rb) are all crucial gatekeepers for oncogene-induced transformation^9–11^. p53 functions as a transcription factor involved in apoptosis, cellular quiescence and senescence^10–12^. PTEN plays a critical role in Akt dephosphorylation which induces G1 cell cycle arrest and apoptosis, along with the regulation of cell adhesion, migration, and differentiation^13, 14^. PTEN can antagonize the PI3K pathway by converting phosphatidylinositol 3, 4, 5-trisphosphate (PIP3) to phosphatidylinositol 3, 4-bisphosphate (PIP2), thereby leading to an increase in PIP2 and inactivation of Akt. The downstream pathway of PI3K, Akt/mTOR, plays an important role in several signaling pathways that enhance tumorigenesis through the coordinated phosphorylation of target proteins to directly regulate cell growth, proliferation, protein synthesis, cell cycle progression, survival, and metabolism^15, 16^. mTOR is a serine/threonine kinase that exists as two distinct multiprotein complexes, mTOR complexes I and II (mTORC1 and mTORC2)^16^. These two complexes differ in their composition, functions, regulation, and responsiveness to rapamycin. mTORC2 is a hydrophobic motif kinase for Akt and shares mTOR and mLST8 with mTORC1. mTORC1 consists of mTOR and raptor (regulatory associated protein of mTOR), whereas mTORC2 comprises mTOR and rictor (rapamycin-insensitive companion)^16^.

ARVs are important pathogens of domestic poultry and wild avian species, causing several diseases including viral arthritis, chronic respiratory diseases, and malabsorption syndrome^17^. Two ARV proteins, p17 and σA, have been detected within the nucleus of infected or transfected cells^18–20^. The p17 protein has been demonstrated to be a nucleocytoplasmic shuttling protein^18^. Our team has demonstrated that p17 modulates several cellular signaling pathways and interacts with several cellular proteins to cause translation shutoff, cell cycle arrest, and autophagosome formation, all of which enhance virus replication^20–24^. Nevertheless, the precise mechanisms of ARV-mediated Akt inhibition are still mostly unknown. In this work we have undertaken a comprehensive investigation to study whether and how the p17 and σA proteins of ARV cooperatively inactivate Akt. We have applied a comparative shotgun proteomics approach using stable-isotope dimethyl labeling and two-dimensional liquid chromatography-tandem mass spectrometry (2D-LC-MS/MS) to obtain a comprehensive overview of ARV-regulated expression of ribosomal and proteasomal proteins. Interestingly, we found that upon ARV infection the levels of most of the 60S and 40S ribosomal proteins are decreased while PSMB6, one of the components of the 20S proteasome, is dramatically increased. This study provides novel insights into ribosomal protein degradation and activation of the proteasome by collaboration of the p17 and σA proteins of ARV via activation of E3 ligase MDM2 and the proteasome PSMB6 subunit. Another compelling finding in this study is that p17 functions as an inhibitor of the CDK2/cyclin A2 complex, further enhancing inhibition of Akt phosphorylation at S473, which reduces the interaction of Beclin 1 with 14-3-3 as well as Beclin 1 and vimentin. The findings presented in this study provide mechanistic insights into the molecular mechanisms of p17 and σA cooperation, which downregulates Akt by suppressing complexes of mTORC2 and CDK2/cyclin A2 as well as activation of the p53/PTEN pathway^23^. Collectively these findings may provide mechanistic insights into how viral infection causes host translation shutoff and cell cycle arrest and induces autophagy to benefit viral replication as observed in earlier reports^22–24^.

## Results

### Large-scale analysis of ribosomal and proteosomal proteins in ARV-infected cells by multidimensional protein identification technology

We previously demonstrated that inhibition of proteasomes by MG132 reduced ARV replication in cultured cells^25^. We next wanted to explore the mechanisms underlying proteasome-mediated ARV replication. To comprehensively monitor the ribosomal and proteasomal protein expression profiles of ARV-infected Vero cells, a comparative shotgun proteomics approach was utilized. Tryptic peptides derived from Vero cells with and without ARV infection were differentially labeled using a stable-isotope dimethyl labeling approach^26^, and the combined mixture was separated and analyzed by two-dimensional liquid chromatography (strong ion-exchange and reversed phase) coupled with tandem mass spectrometry (2D-LC-MS/MS)^27^. Using this approach, up- and down-regulated proteins in ARV-infected Vero cells were identified and quantified simultaneously (Table S1). The expression levels of most 60S and 40S ribosomal proteins and ribosome associated proteins decreased in ARV-infected cells. Interestingly, proteasome subunit beta type-6 (PSMB6), one of the components of the 20S proteasome, was increased more than two-fold (Table S1).

### p17-mediated reduction of ribosomal proteins occurs via the E3 ligase MDM2 to mediate ribosomal protein polyubiquitylation

**Figure 1:**
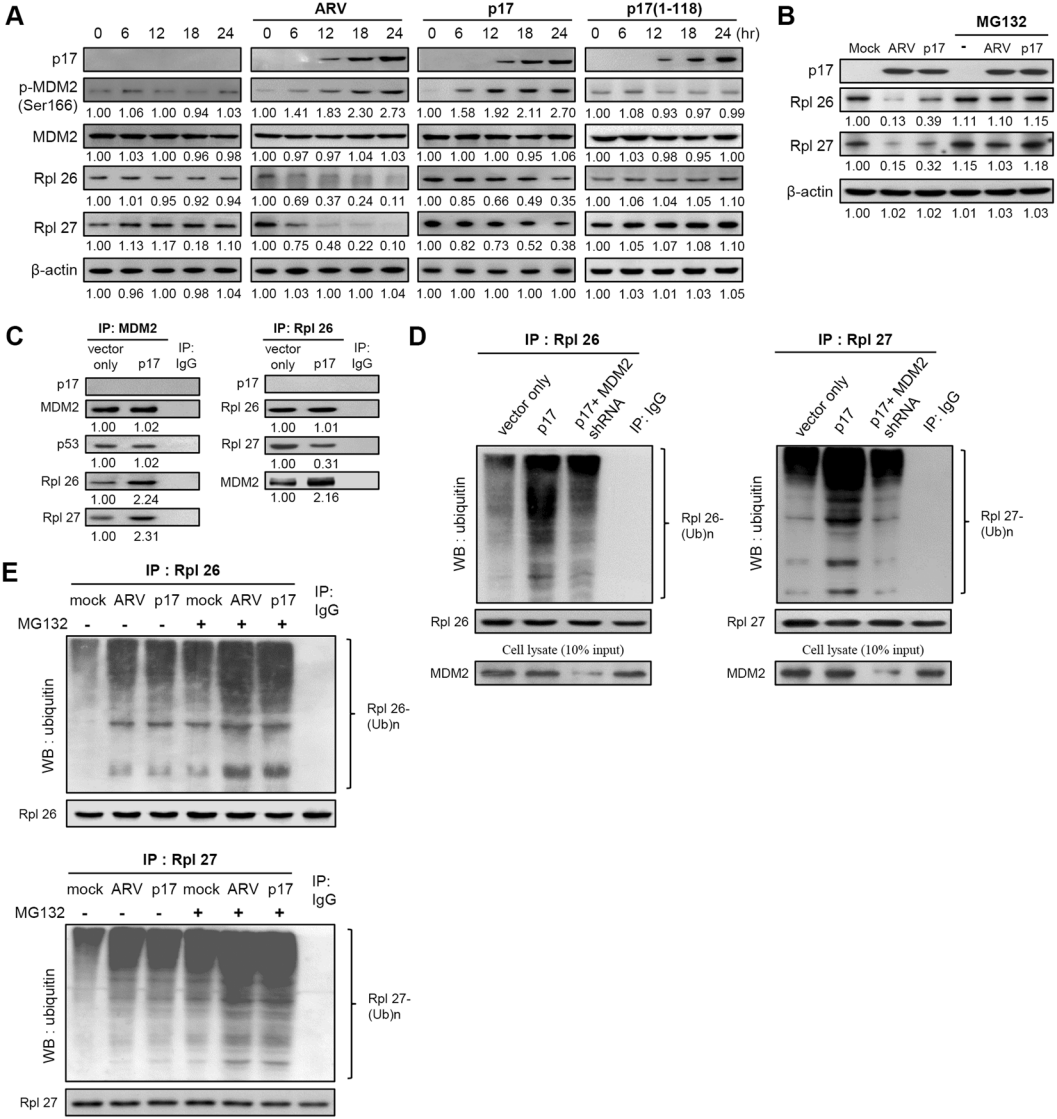
p17 promotes E3 ligase MDM2 targeting to ribosomal proteins. (**A**) Vero cells were infected with ARV at an MOI of 10 or transfected with pcDNA3.1-Flag-p17 or pcDNA3.1-Flag-p17(1–118) plasmids for 24 hours, followed by Western blot assays with the indicated antibodies. (**B**) The expression levels of Rpl26 and Rpl27 in ARV-infected and p17-transfected cells were examined in the presence or absence of MG132 (25 uM), respectively. Whole cell lysates were harvested for Western blot assays with the indicated antibodies. The experiments were repeated three times, and representative blots are shown. (**C**) In co-immunoprecipitation experiments, the binding of E3 ligase MDM2 to ribosomal proteins was examinedinp17-transfected Vero cells. Vero cells were transfected with pcDNA3.1-Flag-p17 plasmid and pcDNA3.1-Flag, respectively. Cell lysates were immunoprecipitated with MDM2 or Rpl26 and interacting proteins were detected with the indicated antibodies. (**D**) Vero cells were transfected with pcDNA3.1-Flag-p17 with or without co-transfection with MDM2 shRNA.The interaction of MDM2 with Rpl26 and Rpl27 was examined. (**E**) Vero cells without treatment or pretreated with MG132 followed by mock infection, ARV infection, and p17 transfection, respectively. The interaction of ubiquitin with Rpl26 and Rpl27was examined. Similar results were obtained in three independent experiments. The protein levels were normalized to those for β-actin. The levels of indicated protein in the mock control or at 0 h were considered 1-fold.The activation and inactivation folds indicated below each lane were normalized against values for the mock control or at 0 h. The uncropped blots with molecular weights are shown in Fig. S5.

Although it has been demonstrated that p17 mediates inactivation of Akt^23^, the precise mechanisms remain largely elusive. The proteomic analysis results inspired us to further explore whether both ribosomal proteins and PSMB6 play critical roles in regulation of mTORC2 and Akt. To investigate whether and how viral proteins downregulate and upregulate these proteins, Western blot assays were performed to analyze the levels of these proteins in cells overexpressing viral proteins p17 or σA. Overexpression of these proteins has been detected within the nucleus of infected and transfected cells^18–20^. As shown in Fig. 1A, a robust reduction in the levels of 60S ribosomal proteins (Rpl 26 and 27) was observed in ARV-infected and p17-transfected cells while the phosphorylated MDM2 (S166) level was elevated in a time-dependent manner. The levels of ribosomal proteins (Rpl 26 and Rpl 27) and p-MDM2 in mock-infected and p17(1–118) mutant-transfected Vero cells did not show significant expression changes over the same time course (Fig. 1A). Since we have demonstrated that the p17 (1–118) mutant protein cannot reach the nucleus to exert modulation on the Tpr/p53/PTEN/Akt pathway^23^, this p17 mutant was used as a negative control. Next we sought to examine whether p17 affects the mRNA levels of these ribosomal proteins and MDM2. Our results revealed that the transcript levels of these proteins were not affected by p17 (Fig. S1A). Several kinases have been shown to be involved in MDM2 phosphorylation of S166^28^. As Akt was proposed to be one of the kinases for S166 phosphorylation of MDM2, Akt was knocked down with an shRNA to study whether Akt is involved in MDM2 phosphorylation. As shown in Fig. S1B, the level of p-MDM2 (S166) was reduced by about 29% in Akt-depleted cells, and p17-mediated phosphorylation of MDM2 could be partially reversed, suggesting that Akt is one of the kinases for S166 phosphorylation of MDM2. Other kinases that are involved in MDM2 phosphorylation will be explored in future studies.

We next wanted to examine whether p17 promotes ubiquitin-proteasome- mediated ribosomal protein degradation. Proteasome inhibitor MG132 was used to block proteasome activity. In the presence of MG132, the levels of ribosomal proteins (Rpl 26 and 27) were not reduced in ARV-infected and p17-transfected cells (Fig. 1B, lanes 5–6), further confirming that p17 mediates ribosomal protein degradation by the ubiquitin-proteasome pathway. Since elevation of p-MDM2 (S166) was seen in p17-transfected cells, we speculated that the reduction in the level of ribosomal proteins was due to the E3 ligase MDM2 triggered by p17 to mediate ribosomal protein polyubiquitylation. To test this, we carried out reciprocal co-immunoprecipitation assays to examine the amounts of MDM2 or ubiquitin binding to ribosomal proteins in p17-transfected cells. As shown in Fig. 1C, p17 robustly enhanced MDM2 targeting to ribosomal proteins. An interesting finding in this study is that the amount of MDM2-p53 association was not increased in p17-transfected cells as compared to the vector only. In addition, p17 neither associates with MDM2 nor ribosomal protein (Rpl 26) (Fig. 1C). An earlier study has demonstrated that ribosomal proteins bind to MDM2 and block MDM2-mediated p53 ubiquitination and degradation, resulting in p53-dependent cell cycle arrest^29^. This may explain why the amount of MDM2-p53 association was not altered and further elucidates the mechanism for how p17 causes p53-dependent cell cycle arrest in several cell lines^20, 23, 24^. Our previous studies have also demonstrated that ARV infection and p17 transfection induce p53 phosphorylation at multiple sites^30^, impairing the ability of MDM2 to target p53. This notion is supported by a previous report showing that phosphorylation of p53 alleviates inhibition byMDM2^31^.

The amount of ubiquitin binding to ribosomal proteins (Rpl 26 and 27) in p17-transfected cells was dramatically increased (Fig. 1D). When we compared the accumulation of ubiquitin and ribosomal proteins in the absence or presence of MDM2 shRNA in cells overexpressing p17 protein, we found that in the presence of MDM2 shRNA, the amount of ubiquitin targeting to ribosomal proteins was dramatically reduced (Fig. 1D, left and right panels; lane 3), demonstrating that MDM2 is is a major E3 ligase for ribosomal proteins. Furthermore, in the absence of the proteasome inhibitor MG132, accumulation of ubiquitin and ribosomal proteins was dramatically decreased in ARV-infected and p17-transfected cells compared to the presence of MG132 (Fig.1E, left and right panels, lanes 1–3). Conversely, in the presence of MG132, a significant accumulation of ubiquitin and ribosomal proteins was observed in ARV-infected and p17-transfected cells, respectively, as compared to controls (without MG132 treatment) (Fig. 1E, left and right panels, lanes 5–6). Taken together, these results suggest a mechanism whereby p17 downregulates the levels of ribosomal proteins through the E3 ligase MDM2 to mediate ribosomal protein polyubiquitylation for protein degradation.

### ARV σA protein increases the proteasome activity and expression level of PSMB6

**Figure 2:**
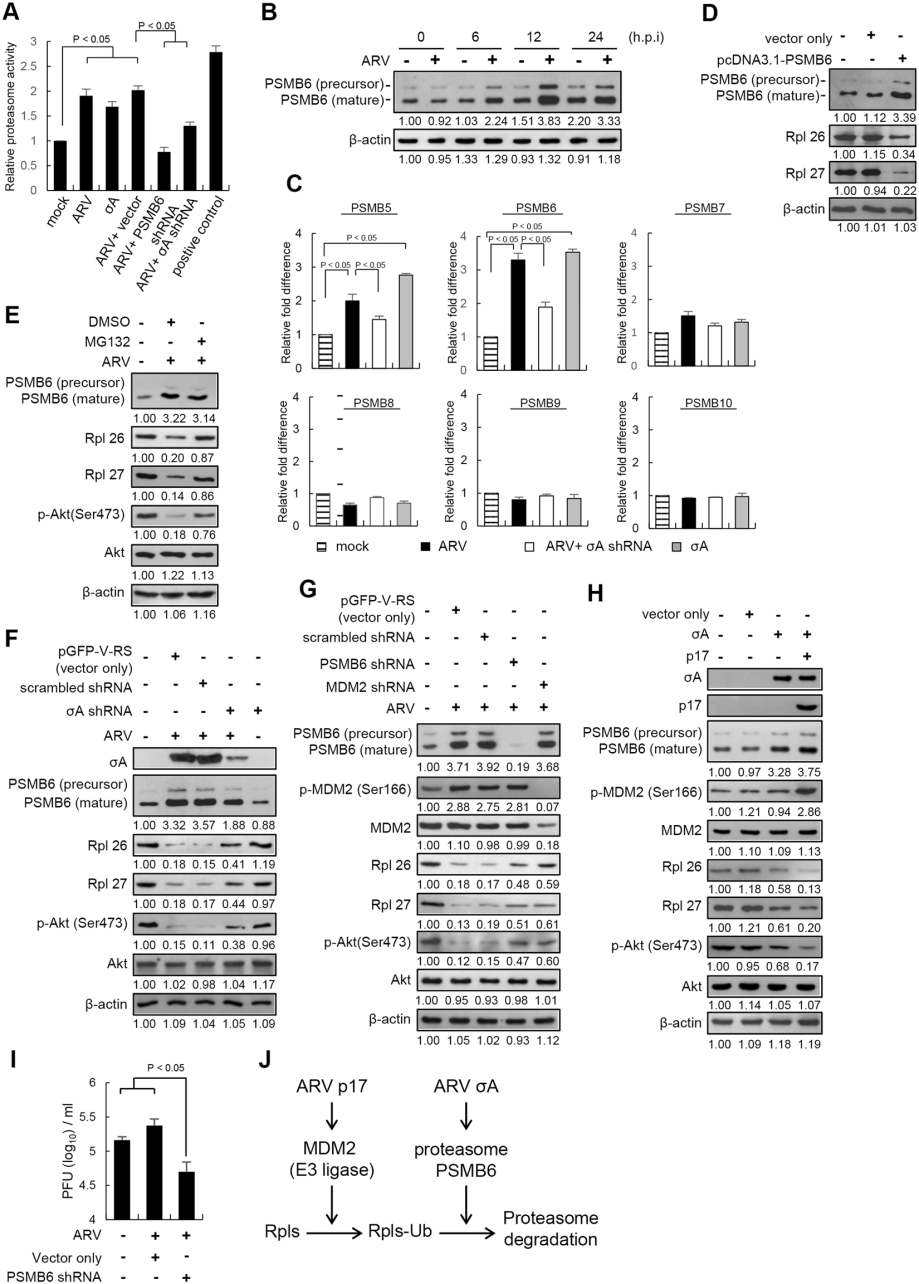
ARV σA protein enhances the proteasome activity and expression level of PSMB6. (**A**) Vero cell lysates from ARV-infected, σA-transfected, and PSMB6- depleted cells with ARV at an MOI of 10 for 24 hours were used to quantify relative proteasome activity. (**B**) Examination of the PSMB6 levels. Vero cells were infected with ARV at an MOI of 10 at indicated time points, followed by Western blot assay with an anti-PSMB6 antibody. (**C**) Analysis of mRNA levels of PSMB6 and other subunits by real-time RT-PCR.The data reveal that PSMB6 is transcriptionally upregulated by σA. (**D**) To confirm whether PSMB6 mediates ribosomal protein ubiquitin-proteasome-mediated degradation and inhibits Akt phosphorylation at S473, Vero cells were transfected with plasmids overexpressing PSMB6 followed by Western blot assay with the indicated antibodies (**E**) In the presence of MG132, the decrease in the levels of p-Akt (S473) in ARV-infected vero cells could be reversed in ARV-infected cells. (**F**) The levels of PSMB6, Rpl26, Rpl27, and p-Akt (S473) were examined in ARV-infected cells co-transfected with an shRNA against σA. (**G**) To confirm whether both PSMB6 and MDM2 mediate ribosomal proteins degradation, knockdown of either PSMB6 or MDM2 with shRNAs was performed followed by Western blot analysis with the indicated antibodies. (**H**) Vero cells were transfected with pcDNA3.1-p17 or co-transfected with pcDNA3.1-p17 and pcDNA3.1-σA plasmids for 24 hours, respectively, followed by Western blot assays with the indicated antibodies. (**I**) Individual 24-well plates of Vero cells were transfected with a PSMB6 shRNA for 24 hours, followed by ARV infection at an MOI of 5 for 24 hours. The ARV-infected cell supernatant was collected at 24 hpi for determining the virus titer. All data shown represent the mean ± SD calculated from three independent experiments. (**J**) A model depicting the cooperation between p17 and σA proteins of ARV to trigger ribosomal protein degradation is shown. The protein levels were normalized to those for β-actin. The levels of indicated proteins in the mock control or at 0 h were considered 1-fold. The activation and inactivation folds indicated below each lane were normalized against against values for the mock control or at 0 h. The uncropped blots with molecular weights are shown in Figs S5 and S6.

As shown in Fig. S2, the expression level of PSMB6 was elevated more than three-fold in ARV-infected cells and σA-transfected cells, but an increase of only 1.32-fold was observed in p17-transfected Vero cells, suggesting that σA protein is a major protein for upregulaing PSMB6. Next we wanted to examine whether σA protein enhances proteasome activity. Interestingly, proteasome activity was significantly increased in ARV-infected and σA-transfected cells (Fig. 2A), but was reversed in cells expressing an shRNA targeting σA, suggesting that σA is the major viral protein responsible for activating proteasome activity. The increase in proteasome activity could be reversed in cells expressing an shRNA targeting PSMB6. This suggests that the increased proteasome activity is related to upregulation of PSMB6. In comparison with uninfected cells, the expression levels of PSMB6 were elevated in ARV-infected cells in a time-dependent manner as revealed by Western blot assays (Fig. 2B), which was consistent with the results presented in Table S1. Analysis of PSMB6 mRNA levels by reverse transcription and real-time PCR in σA-transfected or ARV infected cells corresponds with a 3–4 fold increase in the level of PSMB6 (Fig. 2C), suggesting that PSMB6 is transcriptionally upregulated by σA. PSMB5 was increased 2–3 fold in the same cells, while PSMB7 showed only a small increase (1.5-fold). Other immunoproteasomal subunits (PSMB 8, 9, 10) were unchanged or showed a slight decrease in mRNA levels (Fig. 2C). Furthermore, overexpression of PSMB6 resulted in a three- or four-fold decrease in the levels of Rpl26 and Rpl27 proteins, respectively (Fig. 2D) as compared to the mock control. In the presence of MG132, the decrease in the levels of Rpl26, Rpl27, and p-Akt (S473) in ARV-infected Vero cells could be reversed (Fig. 2E). To further confirm whether σA plays an important role in mediating Rpl26 and Rpl27 protein degradation via activation of PSMB6, depletion of σA with an shRNA was carried out in ARV-infected cells. Our results reveal that the reductions in levels of PSMB6, Rpl26, Rpl27, and p-Akt (S473) could be partially reversed in σA-depleted cells (Fig. 2F, lane 4). Collectivelly, our results suggest that σA plays an important role in upregulation of PSMB6 and downregulation of Rpl26 and Rpl27.

### p17 and σA proteins of ARV cooperate to positively regulate ubiquitin-proteasome-mediated ribosomal protein degradation

Having shown that σA upregulates PSMB6 and p17 promotes MDM2 targeting to ribosomal proteins, both of which reduce the levels of ribosomal proteins, we next wanted to explore whether and how σA cooperates with p17 to regulate ribosomal protein degradation. As demonstrated above, the expression levels of 60S ribosomal proteins Rpl26 and Rpl27 as well as p-Akt (S473) were decreased in ARV-infected cells but were at least partially restored when either PSMB6 or MDM2 was knocked down by shRNAs (Fig. 2G, lanes 4–5), suggesting that both PSMB6 and MDM2 play critical roles in mediating Rpl26 and Rpl27 degradation. This decrease in the levels of Rpl26 and Rpl27 was also enhanced in Vero cells co-overexpressing the σA and p17 proteins (Fig. 2H, lane 4), suggesting that coexpression of σA and p17 proteins has a synergistic effect on downregulation. In addition, knockdown of PSMB6 decreased virus yield (Fig. 2I). Figure 2J depicts how collaboration of these two viral proteins facilitates ubiquitin-proteasome- mediated ribosomal protein degradation.

### p17 reduces mTORC2 assembly

**Figure 3:**
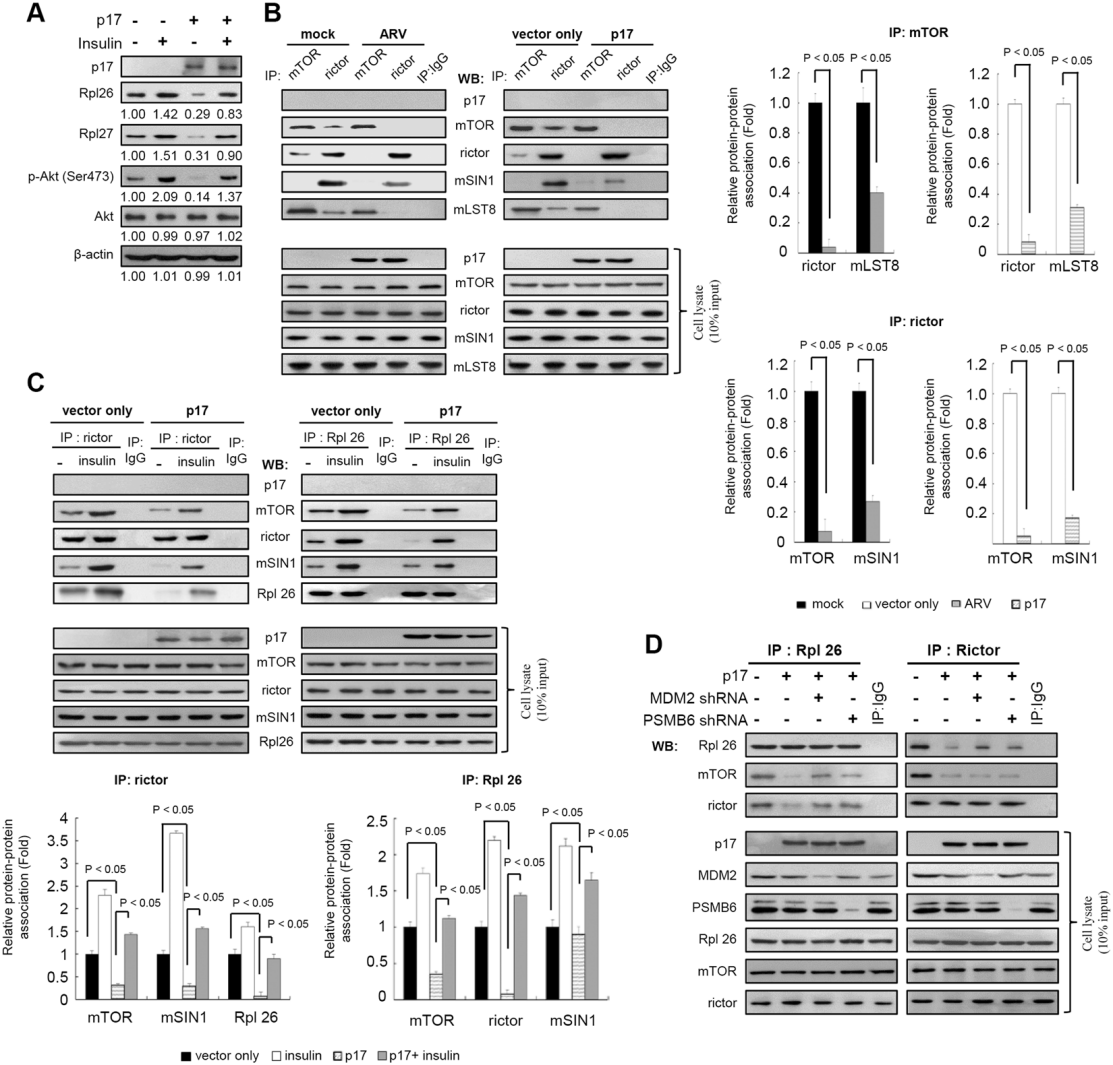
p17 deregulates mTORC2 assembly. (**A**) To study the effect of insulin on ribosomal proteins and Akt phosphorylation at S473, cells were pretreated with insulin (0.2 μm) for 1 hour, followed by transfection with pcDNA3.1-Flag-p17 for 24 hours at 37 °C. Whole cell lysates were harvested for Western blot assays with the indicated antibodies. The protein levels were normalized to those for β-actin.The activation and inactivation folds indicated below each lane were normalized against values for the mock control. those at mock. The levels of indicated proteins in the mock control were considered 1-fold. (**B**) Upper panel: in co-immunoprecipitation experiments, the binding of rictor, mSN1, and MlST8 to mTOR was examined in ARV-infected or p17-transfected Vero cells. Cells were mock-infected or infected with ARV at an MOI of 10 and transfected with either pcDNA3.1-Flag-p17 or pcDNA3.1-Flag (vector only) plasmid for 24 hours. The immunoprecipitated proteins were separated by SDS-PAGE followed by Western blot analysis, and proteins were detected with the indicated antibodies. Lower panel: Data were obtained in three independent experiments, error bars indicate the means ± SD. (**C**) Upper panel: for analysis of the effect of insulin on mTORC2, cells were treated with insulin (0.2 uM) for 1 hour, followed by transfection of cells with either pcDNA3.1-Flag-p17 or pcDNA3.1-Flag plasmid for 24 hours at 37 °C. The interaction of mTOR and rictor with Rpl7 and Rpl26 was examined by co-immunoprecipitation experiments described in panel A. Lower panel: Data shown represent the mean ± SD calculated from three independent experiments. (**D**) To study whether MDM2 and PSMB6 affect the association of mTORC2 and ribosomal proteins, depletion of MDM2 and PSMB6 with shRNAs was performed. The uncropped blots with molecular weights are shown in Figs S6 and S7.

To investigate the effect of insulin on ribosomal proteins and Akt phosphorylation at S473, cells were pretreated with insulin (0.2 μm) for 1 hour, followed by transfection with pcDNA3.1-flag-p17 for 24 hours at 37 °C. The data presented in Fig. 3A indicated that insulin is able to reverse p17-mediated suppression of Rpl26, Rpl27, and p-Akt (S473). As demonstrated above, p17 promotes MDM2 targeting of ribosomal proteins for polyubiquitylation. Thus, we next wanted to further investigate how and whether p17 regulates mTORC2 assembly and the association of mTORC2 and ribosomal proteins. Immunoprecipitation of mTOR using an anti-mTOR antibody revealed that both rictor and mLST8 binding to mTOR were reduced in ARV-infected cells relative to mock controls (Fig. 3B). Similar trends were also observed in p17-transfected cells (Fig. 3B). In reciprocal experiments cell lysates were immunoprecipitated with rictor and proteins were detected by Western blot assay. Immunoprecipitation of rictor using an anti-rictor antibody revealed that the amounts of mTOR-rictor or mSIN1-rictor association in either ARV-infected or p17-transfected Vero cells were significantly reduced as compared to mock treatments (Fig. 3B).

Having demonstrated that p17 affects mTORC2 assembly, we next wanted to examine whether p17 disrupts mTORC2-ribosome association. Previous studies have shown that regulation of the mTOR pathway by growth factors such as insulin is mediated via the PI3K/Akt pathway^6^ and that insulin-mediated activation of class I PI3K leads to production of PIP3 at the inner face of the cell membrane followed by recruitment and activation of Akt through cooperative phosphorylation by PDK1 and mTORC2^32^. In parallel experiments, insulin was used to treat cells to activate mTORC2 as a positive control. Our results indicate that insulin enhances the amount of Rpl26 binding to mTOR, rictor, and mSIN1, respectively, as well as rictor binding to Rpl26 relative to mock treatments (Fig. 3C). In contrast to insulin, p17 reduced Rpl 26 binding to mTOR and rictor (Fig. 3C). This study demonstrates that p17 reduces the assembly of mTORC2 and inhibits mTORC2-ribosome association, both of which in turn impact mTORC2 kinase activity, as seen in the significant decrease in the phosphorylation of mTORC2 substrate (Akt S473) (Fig. 2F,G,H) in both ARV-infected and p17-transfected cells. Work presented by Akcakanat and colleagues suggested that rictor phosphorylation is modulated by mTOR or by one of its downstream targets^33^. As shown in Fig. S3A, dephosphorylation of rictor is seen in both p17-transfected Vero and DF-1 cells. Thus, dephosphorylation of these proteins is very likely related to mTOR inhibition.

Furthermore, immunoprecipitation assays revealed that p17 co-precipitated with neither mTOR nor rictor, suggesting that p17 does not form a stable complex with mTORC2. Furthermore, depletion of either MDM2 or PSMB6 reversed the effects of p17-mediated disassociation of rictor-Rpl26 and mTORC2-Rpl26, but not mTOR-rictor (Fig. 3D). When taken together, these results suggest that the ARV p17 and σA proteins act cooperatively to govern ubiquitin-proteasome-mediated Rpl26 and Rpl27 degradation and suppress Akt phosphorylation at S473.

### p17 inactivates Akt via inhibition of CDK2/cyclin A2 complex by binding to and inhibiting its kinase activity

**Figure 4:**
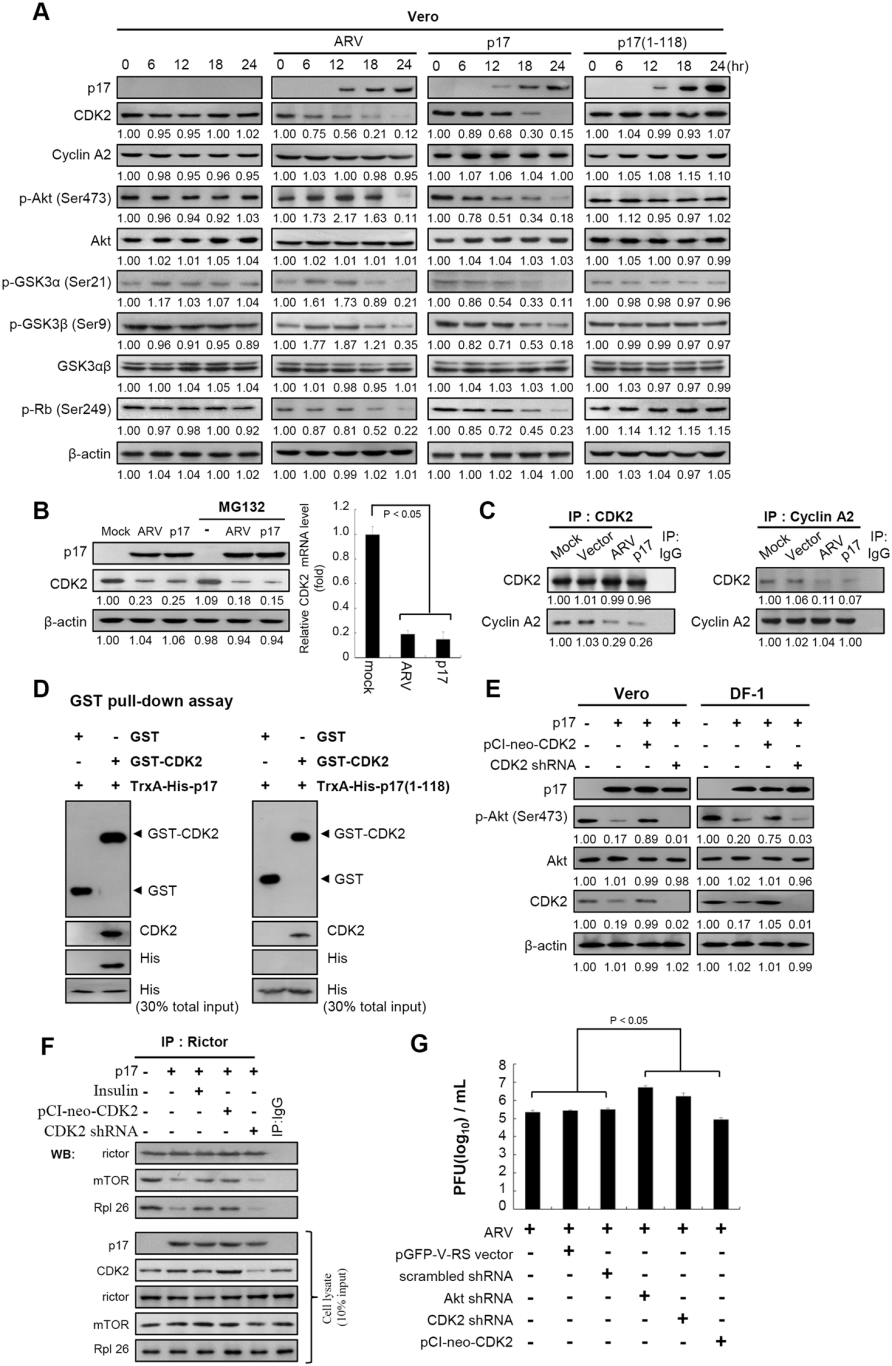
p17 interferes with the formation of the CDK2/cyclin A2 complex, which impedes Akt phosphorylation. (**A**) Levels of CDK2, cyclin A2, p-Akt (S473), p-GSK3α (S21), p-GSK3β (S9), and p-Rb (S249) in ARV-infected and p17-transfected Vero cells were examined. Cells were collected at the indicated points, and whole cell lysates were harvested for Western blot assays. p17 (1–118)-transfected and mock-infected cells were used as negative controls. β-actin was included as a loading control. (**B**) The level of CDK2 was examined in Vero cells without treatment or pretreated with MG132 followed by mock infection, ARV infection, and p17 transfection, respectively. Levels of CDK 2 mRNA in ARV-infected and pcDNA3.1-flag-p17-transfected Vero cells were analyzed by semi-quantitative RT-PCR. Mock infection (cells alone) was used as a negative control. The graph represents the mean ± SD calculated from three independent experiments. (**C**) The amount of CDK2 and cyclin A2 association were examined in either ARV-infected or p17-transfected Vero cells. (**D**) An *in vitro* GST pull-down assay was carried out. Elution fractions were boiled and examined by Western blot analysis. 30% total input of TrxA-His-17 or TrxA-His-17(1–118) mutant represented the internal loading control. (**E**) To confirm whether CDK2 phosphorylates Akt, knockdown of CDK2 with an shRNA and overexpression of CDK2 in p17-transfected cells were carried out, followed by Western blot analysis with indicated antibodies. For negative controls, cells were transfected as indicated. (**F**) To test whether insulin and CDK2 overexpression counteract the inhibitory effect of p17 on mTORC2 complex association, Vero cells were pretreated with insulin (0.2 μm) or transfected with pCI-neo-CDK2 plasmid for 3 hours, respectively, followed by transfection with pcDNA3.1-Flag-p17 for 18 hours. Vero cells were collected and washed twice in phosphate-buffered saline (PBS) and scraped in 200 μl of CHAPS lysis buffer. (**G**) To determine the effects of Akt and CDK2 on ARV replication, individual 24-well plates of Vero cells were infected with ARV at an MOI of 5 for 6 hours, followed by transfection with Akt and CDK2 shRNAs or the pCI-neo-CDK2 plasmid for 24 hours, respectively. The ARV-infected cell supernatant was collected at 24 hpi for determining virus titer. All the data shown represent the mean ± SD calculated from three independent experiments. The protein levels were normalized to those for β-actin.The activation and inactivation folds indicated below each lane were normalized against those at 0 h or mock. The levels of indicated proteins in the mock control or at 0 h were considered 1-fold. The uncropped blots with molecular weights are shown in Figs S7 and S8.

In this work we demonstate for the first time that a dramatic reduction in CDK2, p-Akt (S473), p-GSK3α (S21), and p-GSK3β (S9) levels in p17-transfected cells occurs in a time-dependent manner (Figs 4A and S4A). The unexpected finding of the p17-downregulated CDK2 level inspired us to further explore whether p17 inactivates CDK2/cyclin A2 kinase activity. Since p-Rb is the substrate for CDK2, the phosphorylation level of p-Rb was examined. Importantly, the level of phosphorylated p-Rb (S249) was reduced up to nearly five-fold in p17-transfected Vero (Fig. 4A) and DF-1 cells (Fig. S4A) in a time-dependent manner. No changes were observed in p17(1–118) mutant-transfected cells (Figs 4A and S4A). The p17 mutant protein does not reduce the level of Akt phosphorylation at S473. In response to p17-mediated Akt inactivation, the downstream targets of Akt, GSK3α/β, were examined. The phosphorylated forms of GSK3α/β were increasingly reduced to more than five-fold in p17-transfected cells in a time-dependent manner (Fig 4A). These findings are consistent with our previous study^23^. To investigate whether p17 mediates CDK2 protein degradation, the proteasome inhibitor MG132 was used. In the presence of MG132, the levels of CDK2 in either ARV-infected or p17-transfected cells were unchanged (Fig. 4B). Furthermore, RT-PCR analysis suggested that the observed decrease in the CDK2 mRNA level in p17-transfected cells (Fig. 4B) may be due to transcriptional downregulation of CDK2 by p17. As shown in Fig. 4C, the amount of CDK2/cyclin A2 association was reduced in both ARV-infected and p17-transfected Vero cells, as revealed by reciprocal co-immunoprecipation assays. To explore whether p17 binds to and inhibits CDK2/cyclin A2 kinase activity, both GST pull-down and *in vitro* kinase assays were carried out. The integrity of the purified proteins was confirmed by SDS-PAGE and Coomassie brilliant blue staining (Fig. S4B). In this experiment, p17 was efficiently precipitated with GST-CDK2 (Fig. 4D). GST alone did not bind to p17, indicating that the interaction was specific to p17 sequences. Interestingly, deletion of the carboxyl terminus of p17 in p17(1–118) caused a significant decrease in CDK2 interaction (Fig. 4D), suggesting that the carboxyl terminus (aa 119–146) of p17 is required for its interaction with CDK2.

To confirm the observation that the binding of p17 to CDK2 inhibits its kinase activity, an *in vitro* kinase assay using p-Rb as a substrate was performed. The p17(1–118) mutant and BSA were used as negative controls. An increasing concentration of p17 led to a decreased level of p-Rb (S249) phosphorylation (Fig. S4C). The Ki value for inhibition of CDK2/cyclin A2 complex by p17 that affects p-Rb phosphorylation is about 45 nM (Fig. S4C). Neither the p17(1–118) mutant nor BSA inhibited CDK2/cyclin A2 kinase activity (Fig. S4C). This data further confirms that the carboxyl terminus of p17 is critical for CDK2 binding leading to inhibition of CDK2 and Akt kinase activity. This notion is supported by a recent report that the CDK2/cyclin A complex promotes Akt activation by facilitating or functionally compensating for S473 phosphorylation^6^.

A previous study demonstrated that mTORC2 phosphorylates Akt at position S473 in the C-terminal hydrophobic motif, which in conjunction with PDK1-regulated phosphorylation at T308, drives activation of Akt^34^. In this work, the level of phosphorylated Akt at S473 was reduced in CDK2-knocked down Vero and DF-1 cells (Fig. S4D), indicating that CDK2 is involved in regulation of Akt phosphorylation. Futhermore, we found that overexpression of CDK2 reversed the effect of p17-mediated inhibition of phosphorylated Akt at S473 (Fig. 4E, left and right panels, lanes 3). In p17-transfected and CDK2-depleted cells, the level of phosphorylation of Akt S473 was reduced to barely detectable levels (0.03 or less; Fig. 4E, left and right panels, lanes 4). To investigate whether insulin treatment and CDK2 overexpression counteract the inhibitory effect of p17 on mTORC2 complex association, Vero cells treated with insulin or CDK2 overexpression in p17-transfected cells were examined. Our results revealed that insulin and CDK2 overexpression could reverse the inhibitory effect of p17 on mTORC2 complex association (Fig. 4F). Collectively, our previous and current data suggest that p17 impedes Akt phosphorylation by suppressing both mTORC2 and CDK2 kinase activities and by activating the p53/PTEN signaling pathway^23^.

Aside from this, knockdown of Akt and CDK2 with shRNAs enhanced virus yield (Fig. 4G) while overexpression of CDK2 reduced virus yield (Fig. 4G). The result that knockdown of CDK2 increased virus yield is in agreement with our previous studies suggesting that knockdown of either CDK4 or CDK1 with shRNAs increased virus replication^23, 24^ and may allow the virus to access the host replication machinery without competing with cellular DNA replication. Taken together, this study suggests a specific mechanism by which p17 interferes with the formation of the CDK2/cyclin A2 complex by downregulating CDK2 expression level and by direct binding to CDK2, leading to inhibition of CDK2 kinase activity and subsequent inactivation of Akt, which benefits virus replication.

### p17 reduces the association between Beclin 1 and 14-3-3

**Figure 5:**
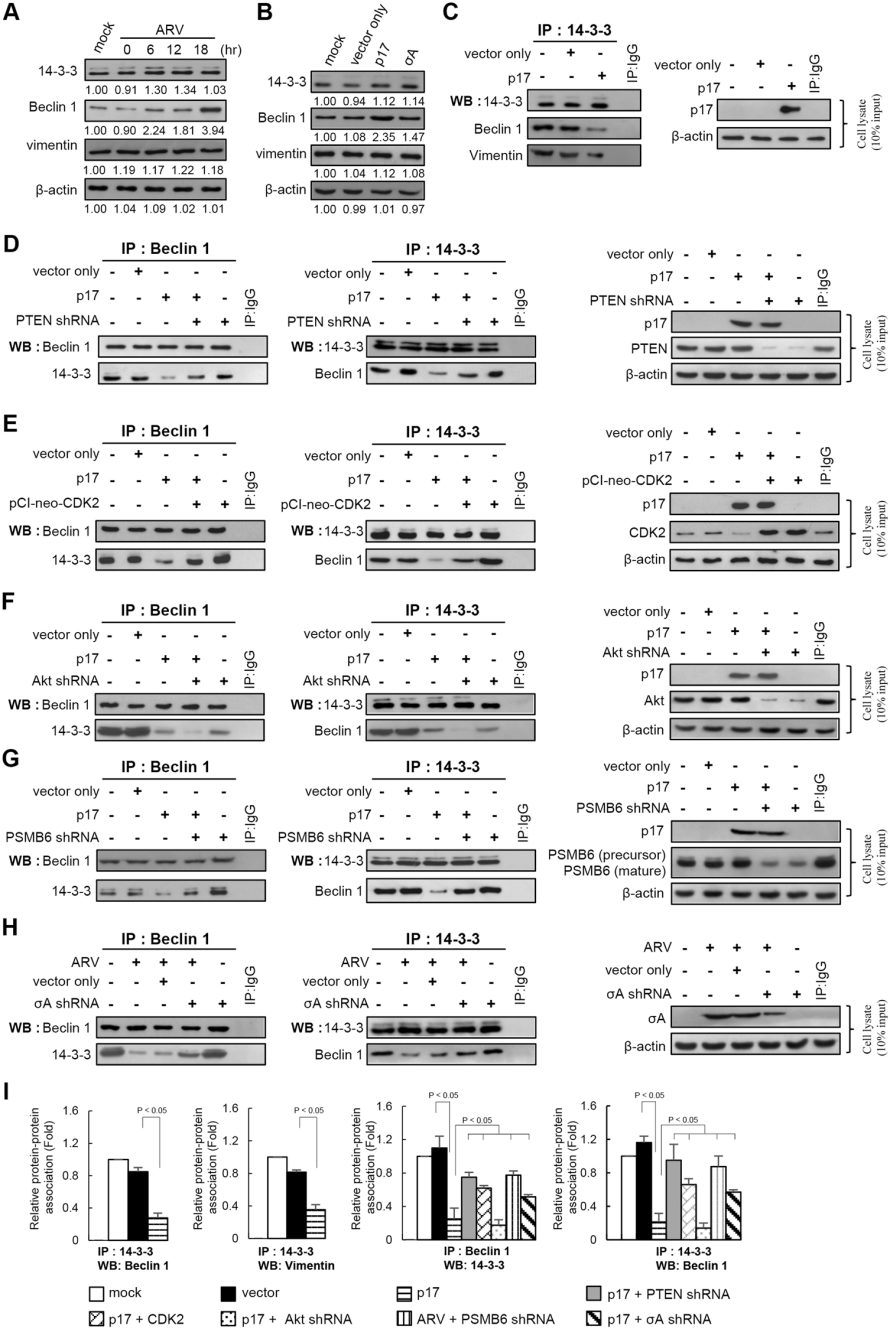
p17 inactivates Akt reducing the association of Beclin 1 and 14-3-3. Vero cells were infected with ARV at an MOI of 10 (**A**) or (**B**) transfected with pcDNA3.1-Flag-p17 or pcDNA3.1-Flag-σA plasmids for 24 hours and protein analyzed by Western blot assays. (**C**) In co-immunoprecipitation experiments, the amounts of 14-3-3-Beclin 1 and 14-3-3-vimentin association were examined in p17-transfected Vero cells. Western blot assays of Beclin 1, 14-3-3 and vimentin contained in 14-3-3 immunoprecipitates were performed. Rabbit IgG was used as a negative control. (**D,E**) In reciprocal co-immunoprecipitation experiments, the binding of 14-3-3 and Beclin 1 was examined in p17-transfected and PTEN shRNA-cotransfected Vero cells (**D**) as well as CDK2 overexpression (pCI-neo-CDK2 vector) in p17-transfected Vero cells (**E**). (**F**) To study whether p17-mediated Akt inhibition leads to an increased amount of Beclin 1 and 14-3-3 association, the amounts of Beclin 1 and 14-3-3 association in Akt-knockdown Vero cells or in p17-transfected or mock-transfected cells were examined. (**G**) In reciprocal co-immunoprecipitation experiments, the binding of 14-3-3 and Beclin 1 was examined in either p17-transfected or PSMB6 shRNA-cotransfected Vero cells. Western blot analysis of 14-3-3 and Beclin 1 contained in 14-3-3 or Beclin 1 immunoprecipates was performed. (**H**) In reciprocal co-immunoprecipitation experiments, the binding of 14-3-3 and Beclin 1 was examined in either ARV-infected or σA shRNA-transfected Vero cells. Western blot analysis of 14-3-3 and Beclin 1 contained in 14-3-3 or Beclin 1 immunoprecipitates was performed. (**I**) Data shown represent the mean ± SD calculated from three independent experiments in distinct shRNA treatments as shown in Fig. 5C–H. The amounts of Beclin1/14-3-3 association were normalized against the value in the negative control. The level of the negative control was considered 1-fold. Data shown represent the mean ± SD calculated from three independent experiments. Controls of knockdown of PTEN, Akt, CDK2, PSMB6, and σA in Fig. 5D,E,F,G,H are shown. The uncropped blots of blots with molecular weights are shown in Figs S8 and S9.

ARV infection and p17 transfection have been implicated in downregulation of Akt and induction of autophagy via activation of the p53/PTEN pathway and inhibition of the Akt/mTORC1 pathway, benefiting virus replication^22, 23^. We next wanted to investigate whether p17-mediated Akt inactivation induces autophagy by directly regulating the core autophagy machinery. We examined the effects of p17 on association of Beclin1/14-3-3 and 14-3-3/vimentin via inactivation of Akt. The amounts of 14-3-3, vimentin, and Beclin 1 in cellular extracts from untreated, ARV-infected and 17- and σA-transfected cells were analyzed by Western blot assays. As shown in Fig. 5A,B, no significant changes in the levels of 14-3-3 and vimentin were observed in ARV-infected and p17-transfected cells in comparison with untreated cells, whereas an increase in the level of Beclin 1 was observed. Co-immunoprecipitation results indicated the Beclin 1 and 14-3-3 association was reduced in p17-transfected cells (Fig. 5C). This reduction was at least partially reversed in PTEN-, PSMB6-, and σA-knock down Vero cells (Fig. 5D,G,H), respectively. Overexpression of CDK2 increased the amount of Beclin 1 and 14-3-3 association (Fig. 5E, left and middle panels, lane 5) and partially reversed p17-mediated reduction of Beclin 1 and 14-3-3 association (Fig. 5E, left and middle panels, lane 4). Furthermore, as PSMB6 acts as a negative regulator in the regulation of the mTOR2-ribosome/Akt pathway, it is interesting to see whether depletion of Akt or PSMB6 affects the Beclin 1 and 14-3-3 association. Depletion of Akt decreased the amount of Beclin 1 and 14-3-3 association (Fig. 5F, left and middle panels, lane 5) and enhanced the p17-mediated reduction of Beclin 1 and 14-3-3 association (Fig. 5F, left and middle panels, lane 4). Figure 5G indicates that shRNA knockdown of PSMB6 increased Beclin 1 and 14-3-3 association as compared to p17-transfected cells. Similar trends were also seen in σA-knockdown and ARV-infected Vero cells (Fig. 5H). These results suggest that ARV promotes PSMB6 to trigger ribosomal protein degradation, which in turn downregulates Akt signaling and induces autophagy. Figure 5I summarizes the data as the mean ± SD calculated from three independent experiments with distinct shRNA treatments as shown in Fig. 4C–H.

### Nuclear targeting of p17 is important for induction of autophagy

**Figure 6:**
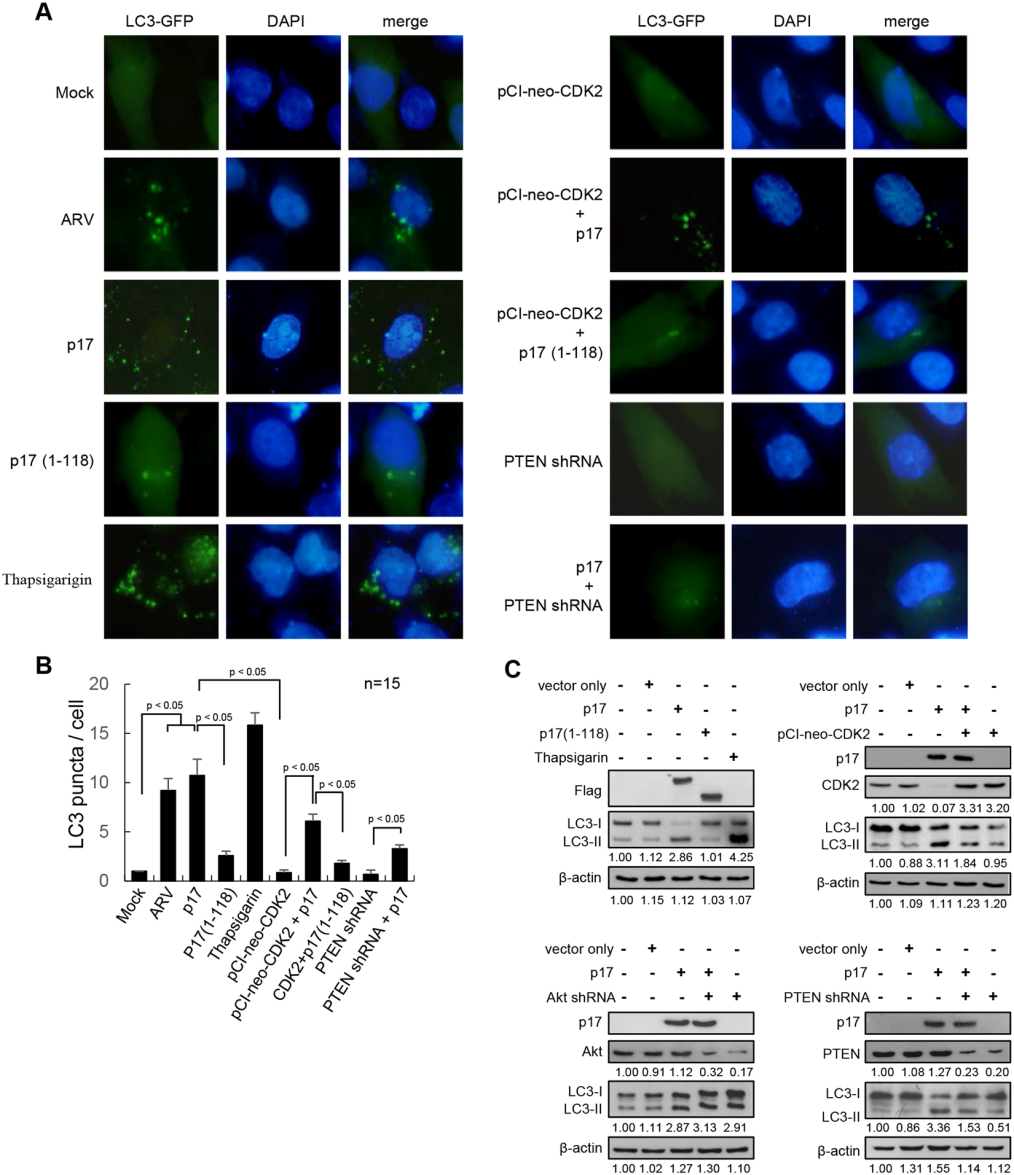
Nuclear import of p17 is important for induction of autophagy. (**A**) A GFP-LC3 plasmid was used to observe LC3 punta in Vero cells under a fluorescence microscope. All conditions for the treated- and untreated-cells are described in the Material and Method section. Scale Bar: 20 μm. (**B**) GFP-LC3 plasmid was applied to observe LC3 punta under a fluorescence microscope. Quantitation results from Fig. 6A represents mean GFP-LC3 puncta per cell. n = 15. (**C**) Levels of LC3-II in different treatments were examined. Whole cell lysates were harvested for Western blot assays. pcDNA3.1-Flag-p17 (1–118)-transfected and mock-infected cells were used as negative controls. β-actin was included as a loading control. The LC3-II level of mock control (cells alone) was considered 1-fold. The activation folds indicated below each lane were normalized against values for the mock control. The uncropped blots with molecular weights are shown in Fig. S9.

Formation of autophagosomes can be analyzed by transfection with a fusion protein of green fluorescent protein with LC3 (GFP-LC3), a fluorescent marker of autophagosomes. The staining of LC3-I appears diffusely in the cytoplasm whereas the staining of LC3-II is punctate^35^. Assays performed in both ARV-infected and p17-transfected Vero cells revealed a significant increase in the numbers of GFP-LC3 puncta (Fig. 6A,B). A similar trend was observed in thapsigargin (TG)-treated Vero cells as compared to the mock control (Fig. 6A). A recent study in our laboratory suggested that nuclear import of p17 negatively regulates nucleoporin Tpr, which in turn activates p53, PTEN and p21 and negatively regulates Akt^23^. Thus, we next wanted to explore whether nuclear import of p17 is important for induction of autophagy. A deletion mutant of p17, lacking the nuclear localization signal (NLS) sequences (aa 119–127), was used^23^. The truncated p17 (dNLS-p17) is not able to enter the nucleus to activate the Tpr/p53/PTEN/Akt pathway^23^, which induces only a few GFP-LC3 punta in comparison to p17-transfected cells (Fig. 6A,B), implying that nuclear targeting is important for p17-induced formation of autophagosomes. Figure 6B summarizes results from Fig. 6A. As shown in Fig. 6C, increased levels of LC3-II were found in TG-treated and Akt knock down cells while decreased levels of LC3-II were found in PTEN knock down cells. Furthermore, overexpression of CDK2 reversed p17-mediated upregulation of LC3-II. The p17 (1–118) mutant protein induces a low level of LC3-II as revealed by Western blot assays. The results are consistent with our previous study^23^ and the data shown in Fig. 6A,B.

Although nuclear targeting of p17 is important for induction of autophagy^22, 23^, p17-induced autophagy is accomplished through multiple signaling pathways such as the p53/PTEN/Akt, AMP-activated protein kinase (AMPK), and dsRNA-dependent protein kinase (PKR)/eIF2α^22^. This might explain why few GFP-LC3 punta were observed in p17(1–118)-transfected cells. In PTEN shRNA-treated cells, a significantly lower number of GFP-LC3 puncta was observed (Fig. 6A), further suggesting that PTEN is the upstream signal for negative regulation of the Akt/mTORC1 signaling pathway which inhibits autophagosome formation.

### p17 inhibits the CDK2/cyclin A2 complex leading to induction of autophagosome formation

As demonstrated in Fig. 5, p17 reduces the association of Beclin 1 and 14-3-3 and in turn increases the functional Beclin 1 level. Since knockdown of CDK2 with an shRNA led to suppression of phosphorylation of Akt S473 (Fig. 4C), it is interesting to determine whether p17 inhibits CDK2 kinase activity, leading to inactivation of Akt and an increase in numbers of GFP-LC3 puncta. Studies with GFP-LC3 have revealed that exogenous expression of CDK2 in Vero cells inhibited significantly the formation of GFP-LC3 puncta (Fig. 6B). As shown in Fig. 6C, overexpression of CDK2 reversed p17-mediated upregulation of LC3-II. Importantly, the negative effect of CDK2 on formation of GFP-LC3 puncta could be reversed in cells overexpressing p17 protein, suggesting that p17 induces autophagosome formation at least in part through inactivation of CDK2 activity. Furthermore, the p17(1–118) mutant which does not inhibit CDK2 kinase activity reversed weakly the effect of CDK2 on formation of GFP-LC3. Taken together, our results reveal that the carboxyl terminus of p17 is crucial for inhibition of CDK2 kinase activity and is necessary for p17-induced autophagy.

## Discussion

The novel discovery that p17 collaborates with σA in inactivating Akt through inhibition of both mTOR2 and CDK2/cyclin A2 complexes and through activation of the p53/PTEN signaling pathway offers novel insights into its functionality in inducing the formation of autophagosomes, which benefits virus replication. In the case of Beclin 1 regulation, Pattingre and colleagues demonstrated that the antiapoptotic protein, Bcl-2, interacts with Beclin 1 and inhibits Beclin 1-dependent autophagy^36^. It has been demonstrated that autophagy and apoptosis are basic cellular pathways that are regulated by JNK-mediated Bcl-2 phosphorylation^37^. JNK-mediated Bcl-2 phosphorylation inhibits its binding to Beclin1, which promotes the formation of autophagosomes^37^. A recent study on caspase activation in cells undergoing autophagy has demonstrated that Beclin 1 can be cleaved by activated caspase to induce apoptosis^38^. More recent evidence suggested that Akt phosphorylates Beclin 1 to enhance its interactions with 14-3-3 and vimentin, thereby suppressing autophagy^8^. The current study and our earlier report suggest that p17 upregulates Beclin 1^22, 39^. This study further provides understanding of the mechanism underlying p17-mediated inhibition of Beclin1 phosphorylation by Akt. Beclin 1 is a subunit in PI3K class III complexes and autophagy induction is dependent on lipid kinase activity of class III PI3K^40, 41^. The precise mechanism by which p17 induces autophagy via regulation of Beclin 1/class III PI3K complexes will be elucidated in the future.

As the cell cycle and proliferation are important in virus replication, proteasomes might act as virulence factors^42^. Our earlier study demonstrated that proteasome inhibition by MG132 resulted in a reduction in ARV replication^25^. To explore the precise mechanism, we have undertaken a comprehensive study to investigate the roles of ribosomal and proteasomal proteins in regulation of mTORC2, Akt, and autophagosome formation. This study demonstrates a specific mechanism whereby ARV coordinately regulates the degradation of ribosomal proteins by p17-mediated activation of E3 ligase MDM2 to target ribosomal proteins and by σA-mediated upregulation of proteasome PSMB6, both of which in turn inactivate mTORC2 and subsequently block Akt-mediated phosphorylation of Beclin 1, thereby inducing autophagy. Relative to mTORC1, little is known about the regulatory inputs to mTORC2. This is the first report that both p17 and σA proteins of ARV work together to inactivate mTORC2. This study offers mechanistic insights into concerted effects of p17 and σA in suppression of mTORC2 and Akt signaling, triggering autophagosome formation by releasing Akt-mediated inhibitory Beclin1 phosphorylation^8^. These findings are in agreement with a recent report that Akt-mediated phosphorylation of Beclin 1 leads to a blockade of autophagy, thereby enhancing its effects in oncogenicity^8^. In addition to p17-mediated inactivation of Akt by activating the Tpr/p53/PTEN signaling pathway^23^ and by suppressing mTORC2, this study describes a novel function for p17 whereby it inhibits the CDK2/cyclin A2 complex kinase activity, leading to Akt inactivation and induction of autophagy. Furthermore, we have identified the carboxyl terminus of p17 as necessary for CDK2 binding and induction of autophagy. This study provides several lines of evidence that p17 impedes multiple phosphorylation events on Akt. The inhibition of Akt by p17 and σA proteins of ARV is dependent on multiple mechanisms: (i) inactivation of Akt by p17 via activation of the Tpr/p53/PTEN signaling pathway^23^, (ii) deregulation of mTORC2-ribosome association by p17 and σA proteins, and (iii) suppression of the CDK2/cyclin A2 complex by binding of p17 to CDK2.

In addition to suppression of mTORC2 by deregulating mTORC2 assembly and by disassociating mTORC2-ribosomes, we also provide two lines of evidence to explain how p17 deregulates mTORC2 assembly. First, p17 reduces the association of mSIN1or mLST8 to rictor as well as rictor or mLST8 to mTOR, resulting in disassociation of mTORC2, accompanied by decreased phosphorylation of Akt S473. The finding of decreased binding of mSIN1 to rictor is consistent with an earlier study that showed that mSIN1 plays an important role in maintaining rictor-mTOR complex and Akt S473 phosphorylation^43, 44^. Several groups have also reported that rictor, mLST8, and mSIN1 each help to maintain the integrity of mTORC2^43–46^. In addition, p17 stimulates dephosphorylation of rictor in cell lines, possibly reducing the affinity of rictor for mTOR^33^. This notion is supported by previous reports that suggest hypophosphorylated rictor has lower affinity for mTOR than the phosphorylated form^33^.

p53 regulates the transcription level of some autophagy modulators in the nucleus, including PTEN^47^. Akt functions downstream of the PTEN/PI3K signaling pathway, that is involved in cancer^38–50^. Our previous study demonstrated that p17 inactivates Akt and its downstream targets through suppression of nucleoporin Tpr and activation of the p53/PTEN signaling pathway to trigger autophagy^23^. p17 is a shuttle protein^18^ but it remains unclear whether its nuclear import is important for autophagosome formation and virus replication. This study provides further clues that nuclear targeting of p17 is an important factor for autophagy induction. Based on our previous^20–24^ and current findings, we suggest that p17-mediated autophagosome formation, translation shutoff, and cell cycle arrest in replication-activated cells may allow the virus to access the host replication machinery without competition with cellular DNA, which could benefit virus replication. The p17 protein shuttles to the nucleus and exerts its effects on regulation of nuclear signaling, Tpr/p53/PTEN signaling, and gene regulation. This may explain why ARV, a cytoplasmic virus, encodes nuclear shuttling proteins. The combination of p17-mediated suppression of complexes of mTOR and CDK2/cyclin A2 and activation of the p53/ PTEN signaling pathway, all of which inactivate Akt, cause cell cycle arrest, and induce autophagy.

**Figure 7:**
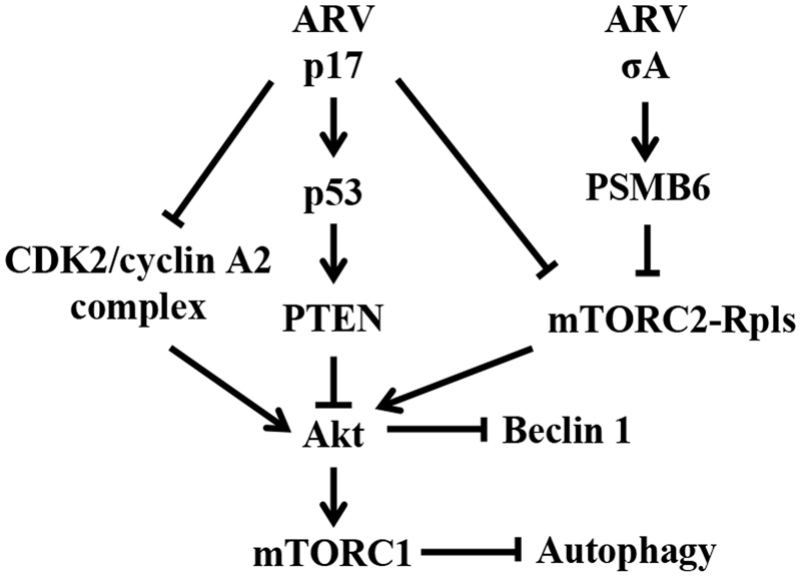
A model depicting the mechanism of avian reovirus p17 protein cooperating with σA to regulate autophagy and the cell cycle via suppression of mTOR and CDK2/cyclinA2 complexes and activation of proteasome subunit PSMB6 to facilitate ribosomal protein degradation. p17-mediated reduction of ribosomal proteins occurs via the E3 ligase MDM2 to mediate ribosomal protein polyubiquitylation. We also uncovered that ARV σA protein increases the proteasome activity and expression level of proteasome subunit PSMB6. Furthermore, p17 binds to and inhibits the CDK2/cyclin A2 complex, further reducing phosphorylation of Akt S473. Both p17 and σA proteins act cooperatively to inhibit mTORC2, which inhibits Akt and activates Beclin, inducing autophagy. The present study provides mechanistic insights into cooperation between p17 and σA proteins of ARV to negatively regulate Akt by downregulation of mTORC2 and CDK2/cyclin A2 complexes and upregulation of proteasome subunit PSMB6, which together induces autophagy.

ARVs appear to have evolved strategies that alter the physiology of host cells during infection to enhance viral replication and suppress the host response to infection in ways that are crucial for the completion of the viral life cycle. A clear understanding of the molecular basis for ARV-host interaction can shed light on normal cellular events and on the specific mechanisms that ARV uses to control its hosts. The model in Fig. 7 illustrates a novel mechanism of cooperation between p17 and σA proteins of ARV to negatively regulate complexes of mTORC2 and CDK2/cyclin A2 and Akt and positively regulate p53, PTEN, and Beclin 1, leading to induction of autophagy.

## Materials and Methods

### Virus and cells

The S1133 strain of ARV was used in this study. African green monkey kidney (Vero) cells and immortalized chicken embryo fibroblast (DF-1) cells were maintained in Dulbecco’s Modified Eagle’s Medium (DMEM) and supplemented with 10% fetal bovine serum (FBS) and 10 mM 4-(2-hydroxyethyl) piperazine-1- ethanesulphonic acid (HEPES) (pH 7. 2). Cells were seeded 1 day before each experiment in 6-cm cell culture dishes with 1 × 10^6^ cells and grown at 37 °C in a 5% CO_2_ humidified incubator. All cells were cultured in serum-free medium for 2 hours followed by refreshing the medium containing 5% of FBS and continuing growth until cell confluence reached about 75%.

### Reagents and antibodies

Insulin was purchased from Life Technologies (Carlsbad, USA). Thapsigargin (TG) which specifically inhibits the fusion of autophagosomes with lysosomes, Rb protein, formaldehyde-D2 (20% solution in D_2_O), iodoacetamide (IAM), N-octyl-D-glucopyranoside (NOG), sodium chloride, sodium cyanoborohydride, sodium dodecyl sulfate (SDS) and trichloroacetic acid (TCA)were purchased from Sigma-Aldrich Co. MG132 was from Calbiochem Co. (San Diego, USA). Acetic acid, acetonitrile (ACN), ammonium bicarbonate (ABC), ammonium hydroxide, 1,4-dithiothreitol (DTT), formaldehyde (37% solution in H_2_O), formic acid (FA) and sodium acetate (NaOAc) were obtained from J. T. Baker (Phillipsburg, NJ, USA). Pierce BCA Protein Assay Kit was purchased from Thermo (Rockford, USA). Sequencing grade modified trypsin was obtained from Promega (Madison, WI, USA). Pierce® micro-spin columns were purchased from Thermo Scientific (Bellefonte, PA, USA). SP Sepharose agarose beads were purchased from GE Healthcare (Uppsala, Sweden).

Polyclonal antibodies against the ARV p17 protein are from our laboratory stock. A specific monoclonal antibody against σA protein of ARV was generated for this study according to procedures described previously^51, 52^. Rabbit anti-MDM2, rabbit anti-MDM2 (S166), mouse anti-CDK2, mouse anti-cyclin A2, mouse anti-GST, mouse anti-ubiquitin, rabbit anti-rictor, mouse anti-PSMB6, and mouse-anti-GSK3α/β antibodies were purchased from Santa Cruz Biotechnology (Dallas, USA). Rabbit anti-mTOR, rabbit anti-mLST8, rabbit anti-PTEN, rabbit anti-p-Akt (S473), rabbit anti-Akt, rabbit anti-p-Akt (S473), rabbit anti-Akt, rabbit anti-p-GSK3α (S21), rabbit anti-p-GSK3β (S9), mouse anti-p53, rabbit anti-p-Rb (Ser249), rabbit anti-Beclin 1, rabbit anti-vimentin, rabbit anti-14-3-3, and rabbit anti-LC3-II antibodies were from Cell Signaling (Danvers, USA). Mouse anti-β-actin antibody was from Millipore (Billerica, USA). Rabbit anti-mSIN1 antibody, rabbit anti-Rpl26, and rabbit anti-Rpl27 antibodies were purchased from Bethyl Laboratories (Montgomery, USA). Anti-mouse IgG (H+L) and anti-rabbit IgG (H+L) antibodies were purchased from KPL (Washington, USA).

### Trypsin digestion of whole cell lysates of Vero cells with and without ARV infection

Proteins (50 μg) obtained from whole cell lysates of either ARV-infected or mock control Vero cells were dissolved in buffer containing 0.7 μL of 1 M DTT, 9.3 μL of 7.5% SDS and 60 μL of deionized water, and the mixture was incubated at 95 °C for 5 min. The samples were then cooled to room temperature and the reduced proteins were alkylated using 8 μL of 0.5 M iodoacetamide at room temperature in the dark room for 0.5 h. Proteins were precipitated in 20% TCA and the resulting protein pellets were washed using 200 μL of deionized water twice and collected after centrifugation (13000 × g) for 10 min. Pellets were dissolved in 100 μL of 50 mM ammonium bicarbonate (pH 8.5) containing 0.1% N-octyl-b-D- glucopyranoside and sequencing grade trypsin (1 μg) was added with digestion at 37 °C for 16 h. The reactions were terminated using 10 μL of 1% formic acid and the resulting tryptic peptides were lyophilized.

### Dimethyl labeling, SCX fractionation, protein identification and quantitation

The tryptic peptides were labeled as described previously^53^. Briefly, 4 μL of 37% formaldehyde was added to the lyophilized peptides in 100 μL of 0.1 M sodium acetate (pH 5–6). The reactions were performed at room temperature with continuous vortexing. After C18 cartridge desalting and SpeedVac lyophilization, the peptide mixture was dissolved in 100 μL of buffer A (5% ACN containing 0.2% FA) and loaded onto SP Sepharose agarose beads (packed in a Pierce® micro-spin column) for strong cation-exchange (SCX) fractionation. The peptide mixture was fractionated by increasing the portion of buffer B (0.5 M NaCl containing 5% ACN and 0.2% FA) in buffer A to give 8 fractions (0%, 1%, 2%, 5%, 10%, 20%, 40% and 100% buffer B, respectively) for the second dimension reversed phase (RP) LC separation and MS/MS analysis. Each SCX fraction was injected into a Surveyor HPLC system (Thermo Scientific, USA) using a 90-min gradient from 5% to 70% ACN (containing 0.1% FA) at 300 nL/min. The peptides separated by HPLC were ionized using a positive nano-ESI source and analyzed using a LTQ Orbitrap (Thermo Scientific) under survey scan mode. The MS and MS/MS parameters were set as: (1) resolution = 60,000 for MS; (2) scan mode = data-dependent MS/MS scan (its triggering threshold = 500 counts); (3) the precursor ion was ranged from m/z 400 to 1600; (4) the normalized collision energy was set at 35. The MS/MS raw data were converted into MGF files using Mascot Distiller v2.3.2.0 (Matrix Science, London, UK) and the database search was performed using the Mascot search engine v2.3 (110808, 531473 sequences; 188463640 residues) (Matrix Science, UK). The parameters for the search engine were set as: (1) the protein database = Swiss-Prot; (2) the taxonomy = *Homo sapiens* (human) (20245 sequences); (3) allowed missed cleavage = 1; (4) the mass tolerances of precursor and product ions = 5 ppm/0.8 Da, respectively; (5) fixed modification = carbamidomethyl (C), Dimethyl (K), Dimethyl (N-term), Dimethyl:2 H(4) (K) and Dimethyl:2 H(4) (N-term); (6) variable modification = oxidation (M); (7) the identified proteins with Mascot scores >the identity threshold were regarded as significant hits (*p* < 0.05). The quantitative analysis was performed using Mascot Distiller v2.3.2.0 with the following parameters: (1) protein ratio type: average; (2) protocol: precursor; (3) peptide threshold: at least identity; (4) sample ratio: from integrated area; (5) integration source: precursor peak area; (6) integration method: Simpsons; (7) Std. Err. Threshold: 999; (8) correlation threshold: 0.7. The relative protein expression ratios were normalized using β-actin as the internal standard and the thresholds for up- and down-regulation were defined as >1.5 and <0.5, respectively.

### Plasmid construction, protein expression, and purification of fusion proteins

The constructs pET32a-p17, pET32a-p17(1–118), pcDNA 3.1-flag-17, and pcDNA 3.1-Flag-17(1–118) (Table 1) were used as described previously^23^. Plasmids used in this study were described previously^23^. For the construction of the pCI-neo-CDK2 plasmid, CDK2 gene fragments were amplified by PCR and digested with *EcoRI* and *SalI* followed by ligation into the same restriction sites of the pCI-neo vector (Promega, Madison, USA). For construction of the pCI-neo-σA construct, the σA-encoding gene of ARV was amplified from the pET32a-S2-all plasmid as described previously^54^ by polymerase chain reaction (PCR) using σA specific primers (Table 1). PCR reactions contained 500 ng of plasmid DNA, 2 μL of 2.5 mM dNTP (MD Bio Inc), 20 μM of forward primer, 20 μM of reverse primer, 0.5 μL of Pfu DNA polymerase (2.5 u) (MD Bio Inc), 5 μL of 10x pfu buffer and adjusted with nuclease-free water to a final volume of 50 μL. PCR was performed under the following conditions: 94 °C for 3 min and 30 cycles of denaturation for 40 s at 94 °C, annealing for 40 s at 55 °C, extension for 90 s at 72 °C followed by a final 5 min extension at 72 °C. The pCI-neo mammalian expression vector and the amplified full-length σA gene of ARV were digested with *Sal I* and *XbaI* restriction enzymes for 2 hours at 37 °C.

**Table 1:** Primers used in this study.

Gene	Accession number	Sequence (5′-3′)	Location	Expected size (bp)
**Primers for cloning**				
p17^a^ (Flag-tagged)	AF330703	F: CGGAATTCATGCAATGGCTCCGCCATACGA (EcoR I)	293–314	441
		R: GCTCTAGATCATAGATCGGCGTCAAATCGC (Xba I)	733–712	
p17^b^ (His-tagged)	AAF45152	F: CCCAAGCTTATGCAATGGCTCCGCCATACGA (Hind III)	293–314	441
		R: CCGCTCGAGTCATAGATCGGCGTCAAATCGC (Xho I)	733–712	
p17 (1–118)^a^ (Flag-tagged)	AF330703	F: CGGAATTCACAATGCAATGGCTCCGCCATACG (EcoR I)	293–313	354
		R: AAACTCGAGTCAGGATTGAGACCCGCCATCCCAATG (Xho I)	646–623	
CDK2^c^	XM_001113345.3	F: GCGGAATTCATGGAGAACTTCCAAAAGGTGGAAAAG (EcoRI)	235–262	897
		R: AAACTCGAGTCAAAGTCCAAGATGGGGTACTGGC (XhoI)	1131–1110	
Cyclin A2^c^	NP_001777	F: AAAGAATTCATGTTGGGCAACTCCGCGCCGGGG (EcoRI)	306–330	1299
		R: GGGCTCGAGTTACAGATTTAGTGTCTCTGGTGGG (XhoI)	1604–1580	
σA^d^	AF104311	F: TCTAGAACGATGGCGCGTGCCATATAC (XbaI)	16–33	1251
		R: GTCGACCTAGGCGGTAAAAGTG GC (SalI)	1266–1249	
PSMB6^a^	NM_001195714	F: AAGCTTACCATGGCGGCCACCCTACTAGCT (Hind III)	19–39	697
		R: GGATCCTCAGGGGGGCGGTAAAGTGGCAAT (Bam HI)	738–715	
CDK2^d^	XM_001113345.3	F: CGGAATTCACCATGGAGAACTTCCAAAAG (EcoRI)	236–253	897
		R: GTCGACTCAAAGTCCAAGATGGGGTAC (SalI)	1132–1110	
**Primers for semi-quantitative RT-PCR**				
RPL 7	NM_001193556	F: TCAATGGAGTAAGCCCAAAG	313–332	246
		R: CAAGAGACCGAGCAATCAAG	558–540	
RPL 26	NM_001193566	F: CATTTCAATGCACCTTCCCAC	80–100	274
		R: GCCTAGTGATAACCACCTTGC	331–353	
MDM2	NM_001266402	F: GTGAATCTACAGGGACGCCATC	945–963	334
		R: GGCCCAACATCTGTTGCAA	1279–1261	
GAPDH	NM_002046	F: CACCACCATGGAGAAGGCTGGGGCTCA	480–506	454
		R:GGCAGGTTTCTCCAGACGGCAGGTCAG	933–907	

^a^PCR products cloned in pcDNA3.1-Flag vector; ^b^PCR products cloned in pET32a vector; ^c^PCR products cloned in pGEX4T-1vector; ^d^PCR products cloned in cloned in pCI-neo vector.

To create the pcDNA3.1-PSMB6 plasmid, the PSMB6 gene of Vero cells was amplified by reverse transcription (RT)-PCR. RT was carried out using oligo dT primer (Genomics Inc, Taiwan). Briefly, RNA template (2 μg of total RNA) prepared from cells was mixed with oligo dT primer (50 ng), and incubated at 72 °C for 10 min. To obtain cDNAs of Vero cells, RT was carried out at 42 °C for 90 min in 25 μL containing 5 μLof RT buffer, 5 μL dNTPs (10 mM), 2 μL of RNA template (2 μg), 0.5 μLof M-MLV reverse transcriptase (200 units), and deionized water to a final volume of 25 μL. The PCR conditions used for PSMB6 amplification were the same as used for σA with the exception of extension at 72 °C for 1 min. Plasmid DNA and the amplified full-length PSMB6 products were cut with both Hind III and Bam HI restriction enzymes for 2 hours at 37 °C. These inserts and vectors were isolated by gel purification and ligation was carried out at 16 °C overnight.

For construction of pGEX4T-1-CDK2 and pGEX4T-1-cyclin A2 plasmids, the CDK2 and cyclin A2 genes of Vero cells were amplified by RT-PCR using oligo dT and specific primer pairs as shown in Table 1. The cDNA fragments representing the full-length CDK2 gene were generated by RT-PCR from total RNA of Vero cells. RT was carried out using oligo dT primer as described above. Unfortunately, after RT-PCR we could not obtain the cyclin A2 cDNA products from Vero cells. Thus, the cyclin A2 cDNA was purchased from Thermo Fisher Co. (USA) for PCR. PCR consisted of 2 μL of cDNA (500 ng), 2 μL of dNTP (2.5 mM), 1 μL of primer pairs, 0.5 μL of Pfu DNA polymerase (2.5 u), 5 μL of 10x Pfu buffer, and deionized water to a final volume of 50 μL. PCR amplification was performed under the following conditions: 95 °C for 5 min, 30 cycles of 95 °C for 1 min, 56 °C for 1 min, 72 °C for 1 min (for CDK2) or 72 °C for 90 s (for cyclin A2) followed by a final 10 min extension at 72 °C. The purified PCR products of both CDK2 and cyclin A2 were cut with the respective restriction enzymes and introduced into the corresponding sites in the pGEX4T-1 vector (GE Healthcare Life Sciences, UK). The correctness of all constructs prepared in this study was confirmed by DNA sequence analysis.

All recombinant plasmids were transformed into *E. coli* BL21(DE3). The transformed *E. coli* cells were grown in Luria-Bertani (LB) broth with 100 ug/ml of ampicillin at 37 °C to an optical density of 0.6 and then induced with 0.4 mM of IPTG for 5 h at 28 °C. C. To obtain soluble forms of GST-tagged fusion proteins, cells were harvested by centrifugation followed by resuspension in lysis buffer [1x phosphate-buffered saline (PBS), 0.2 mM PMSF, 1% Triton X-100, 0.5% Sodium lauroyl sarcosinate]. After sonication, cell suspensions were centrifuged at 12, 000 × g for 20 min at 4 °C. Cell suspensions were changed to 1x PBS with Amicon Ultra 0.5-ml 10k filters (Millipore) by adding the same volume of 1x PBS at least five times. The cells were collected by centrifugation and resuspended in pGEX4T-1 system lysis buffer (1x PBS, 1% Triton X-100, 0.2 mM PMSF) and sonicated. Cell suspensions were then centrifuged at 12, 000 × g for 20 minutes at 4 °C. Each supernatant was applied to a glutathione-Sepharose 4B column (GE Healthcare Bio-Sciences). After washing beads with 1x PBS washing buffer, the GST fusion proteins were eluted from the column with elution buffer (1x PBS, 10 mM reduced glutathione). For the purification of His-tagged p17, His-tagged p17(1–118), and His-tagged fusion proteins, cells were harvested by centrifugation, followed by resuspension in pET system lysis buffer (20 mM Tris-HCl pH 8.0, 300 mM NaCl, 0.2 mM PMSF, 10% glycerol, 5 mM imidazole) and sonicated. Cell suspensions were centrifuged at 12, 000 × g for 20 minutes at 4 °C. Each supernatant was applied to a nickel column. After washing beads with 150 ml washing buffer, TrxA-His-tagged p17 and TrxA-His-tagged p17(1–118) fusion proteins were eluted from the affinity column with elution buffer (20 mM Tris-HCl pH 8.0, 300 mM NaCl, 0.2 mM PMSF, 10% glycerol, 200 mM imidazole). Finally, purified fusion proteins were changed to PBS buffer with Amicon Ultra 0.5-ml 10k filters (Millipore). Samples were stored at −80 °C for further experiments.

To study whether p17, p17(1–118), and CDK2 affect autophagosome formation, LC3 punta were observed under a fluorescence microscope. Two constructs, pcDNA 3.1-Flag-17 and pcDNA 3.1-Flag-17(1–118) were used as described previously^23^. The pCI-neo-CDK2 plasmid (Table 1) was described above.

### Semi-quantitative RT-PCR

To explore whether ARV infection or p17 transfection impact MDM2, Rpl26, and CDK2 gene transcripts, Vero cells were infected with ARV at an MOI of 10 or transfected with pcDNA3.1-flag-p17. All cultures were harvested and lysed at 24 hours postinfection. Total RNA was isolated from the virus-infected or transfected cells. Total RNAs were subjected to semi-quantitative RT-PCR as described previously^23^. The specific primer pairs used in this work are shown in Table 1. RT and PCR were carried out as described above. PCR amplifications were performed under the following conditions: 94 °C for 3 min, 30 cycles of 94 °C for 1 min, 56 °C for 1 min, 72 °C for 1 min followed by a final 10 min extension at 72 °C. The glyceraldehyde-3-phosphate dehydrogenase (GAPDH) gene was used as an internal control for normalization. Products were verified in a 1.2% agarose gel and the sequences were confirmed by DNA sequence analysis.

### Real-time RT-PCR for amplification of proteasomal subunits

The specific primer pairs used in this work are shown in Table S2. Vero cells were transfected with ARV σA or ARV σA shRNA for 24 hours followed by 3 hours of infection with ARV. Total RNA was isolated from the cells using Trizol and total RNAs were subjected to reverse transcription. To obtain cDNAs of the Vero cells, reverse transcription was carried out at 42 °C for 60 min with 2 µl of 1 µg/µl RNA, 5 µl of 10 mM dNTP, 1 µl of 0.5 µg/µl of oligo dT, 1 µl of M-MLV reverse transcriptase (200U/ µl) and 5 µl of 5X RT buffer (Promega Co., USA) and nuclease-free water in a total volume of 25 µl. Target cDNAs of proteasome subunits were further amplified with FastStart Universal SYBR Green Master (Roche Co., Germany) with primers indicated in Table S2. Each reaction contained 5 µl of 50 ng/µl total cDNA, 0.5 μl forward and reverse primers (600 nM) each, 25 µl of 1X FastStart Universal SYBR Green Master (ROX) reagent and 19 µl PCR grade water to a final volume of 50 µl and mixed carefully by pipetting. The following program was used: 50 °C for 2 min, 95 °C for 10 min, 40 cycles of 95 °C for 15 sec, 58 °C for 1 min. Relative quantitation results were analyzed using an ABI Biosystems 7300 Fast Real-Time PCR System (Applied Biosystems/Life Technologies Co., USA).

### shRNAs used in this study

With the exception of the Akt shRNA, all shRNAs containing scrambled negative shRNAs (Table 2) were obtained from OriGene Co. (Rockville, USA) and constructed in the vector pGFP-V-RS (TR30007). Each set containing four different shRNAs targeting the respective genes was evaluated in Vero or DF-1 cells as described previously^22, 23^. The shRNA with the most significant down-regulated effect for a respective gene was selected and used in this study (Table 2). The Akt shRNA was obtained from Academia Sinica, Taiwan. Cells were harvested for immunoprecipitation using the Catch and Release kit (Upstate Biotechnology) according to the manufacturer’s protocol. About 500 μg of cellular proteins were incubated with 4 μg of the respective antibody at 4 °C overnight.

**Table 2:** shRNAs used in this study.

Target gene	Cat. No.	Tube ID	Sequence (5′-3′)	Cell lines/virus
p53	TG320558	GI379451 GI379448	CTCAGACTGACATTCTCCACTTCTTGTTC CAGCCAAGTCTGTGACTTGCACGTACTCC	Vero DF-1
CDK2	TG320291	GI378385	TGGATGCCTCTGCTCTCACTGGCATTCCT	Vero/DF-1
MDM2	TG311529	GI346110	TTGTTTGGCGTGCCAAGCTTCTCTGTGAA	Vero
PSMB6	TG310109	GI340431	GGTAAGGCAGTCCTTTGCCATTGGAGGCT	Vero
PTEN	TG320498	GI379210	CTTGACCAATGGCTAAGTGAAGATGACAA	Vero
σA (S2 gene)	—	—	^591^GCGACGAATCGTACTCAATTA^609^	ARV^54^
Akt	TRCN0000000607	NM_001895	CGTAAACAACACAGACTTCAA	Vero

### Co-immunoprecipitation assays

Co-immunoprecipitation (Co-IP) assays were carried out as described previously^23^. Co-IP assays were performed using a Catch and Release Reversible Immunoprecipitation System (Millipore, USA). Briefly, 6-cm cell culture dishes were seeded with 1 × 10^6^ DF-1 or Vero cells. Eexperiments were initiated in serum-free medium for 2 hours followed by refreshing the medium containing 2% of FBS overnight once cell confluence reached about 75%. By examining the regulation of association of mTORC2-ribosome by MDM2 and PSMB6 as well as p17-mediated inhibition of Beclin1-14-3-3 association, Vero cells were transfected with the respective plasmids for 24 hours at 37 °C. In co-transfection assays, Vero cells were co-transfected with pcDNA3.1-p17 and the respective shRNAs for 24 hours at 37 °C. The cells were collected and washed twice in phosphate-buffered saline (PBS) and scraped in 200 μl of Triton lysis buffer (20 mM Tris-HCl (pH 7.5), 150 mM NaCl, 1 mM Na_2_EDTA, 1 mM EGTA, 1% Triton, 2.5 mM sodium pyrophosphate, 1 mM beta-glycerophosphate, 1 mM Na_3_VO_4_, 1 µg/ml leupeptin; Cell Signaling, Massachusetts,USA) or CHAPS lysis buffer (40 mM HEPES [pH 7.5], 120 mM NaCl, 1 mM EDTA, 10 mM pyrophosphate, 10 mM glycerophosphate, 50 mM NaF, and 0.3% CHAPS). 1000 μg of cellular proteins were incubated overnight with 4 μg of the respective antibodies, mouse IgG, or rabbit IgG according to the procedures provided by the Catch and Release Reversible Immunoprecipitation System (Millipore). Mouse or rabbit IgG were used as a negative control for the Co-IP experiments, and all conditions were the same as other CO-IP experiments. The column was centrifuged at 4000 rpm for 15 seconds to eliminate the non-targeted proteins. After washing the column twice with 1X wash buffer, the column was then entrifuged at 4000 rpm for 15–30 seconds for each wash. Protein bound to the beads was eluted using denaturing elution buffer. The eluted proteins were separated by SDS-PAGE followed by Western blot analysis with the respective antibodies.

To examine whether ARV p17 regulates mTORC2 assembly, co-immunoprecipitation reactions with p17, mTOR, rictor, mSIN1, and mLST8 were carried out. Cells were transfected with pcDNA3.1-Flag-p17 or pcDNA3.1-Flag plasmids and further incubated for 24 hours at 37 °C. The procedures for treatment of cells and for immunoprecipitation were the same as described above. About 1000 ug of cellular proteins was incubated with 4 ug of anti-mTOR, rictor or IgG antibodies at 4 °C overnight. The conditions for the IP:IgG control are the same as those for rictor, mTOR, and Rpls. To analyze the effect of insulin on mTORC2-ribosome association, cells were treated with insulin (0.2 μm) for 1 hour, followed by transfection with pcDNA3.1-flag-p17 and pcDNA3.1-flag plasmid DNA, respectively, and incubation for 24 hours at 37 °C. About 1000 μg of cellular proteins were incubated with 4 μg of rabbit anti-rictor or Rpl26 at 4 °C overnight. The immunoprecipitated proteins were separated by SDS-PAGE followed by Western blot analysis with the following antibodies: p17, mTOR, rictor, mSN1, mLST8, Rpl26, and Rpl7.

### GST pull-down assays

Interactions between p17 and p17(1–118) mutant and CDK2 were investigated by a GST pull-down assay using His-tagged p17, His-tagged p17(1–118), and GST-tagged CDK2. In interaction experiments, a total of 1 ug purified GST protein or GST fusion proteins (GST-CDK2) were coupled to glutathione-Sepharose 4B beads and incubated at 4 °C overnight with 100 ng purified TrxA-His-p17 or p17 mutant proteins in binding buffer (20 mM Tris-HCl, 25 mM NaCl, 10% glycerol, 1 mM DTT, 1 mM EDTA, 1 mM PMSF, and 10 μg/ml cocktail protease inhibitor). The protein bound glutathione beads were then washed five times with binding buffer and eluted with elution buffer (50 mM Tris-HCl, pH 8.0, 10 mM reduced glutathione). Elution fractions were boiled and examined by Western blot analysis. A total of 30% input of TrxA-His-p17 or TrxA-His-p17 represented the internal loading control of input p17.

### *In vitro* kinase assays

To explore the possibility that the interaction of p17 and CDK2 results in inhibition of CDK2 kinase activity, an *in vitro* kinase assay was performed using Rb as a substrate. Rb (2 μM) was incubated with purified GST-cyclin A2 (10 nM) and GST-CDK2 (10 nM) as well as purified TrxA-His-p17 or TrxA-His-p17(1–118) fusion proteins at 37 °C for 30 min in cold kinase buffer (25 mM Hepes, pH 7.4, 25 mM β-glycerophosphate, pH 7.4, 25 mM MgCl_2_, 0.1 mM Na_3_VO_4_, 0.5 mM DTT) that contained 40 μM ATP in a final volume of 25 μl. The level of phosphorylated vimentin was analyzed by Western blot assay with an anti-Rb (S249) antibody.

### Proteasome activity assays

The cellular activity of the 20S proteasome was evaluated by means of the proteasome activity assay kit (Millipore, USA). Vero cells were obtained 3 hours after ARV infection or 24 hours after pCI-σA transfection. In PSMB6-depleted cells, Vero cells were transfected with PSMB6 shRNA for 24 hours and then infected with ARV at an MOI of 10 for 3 hours. Cells were collected by scraping from the culture dish and washed twice with PBS. After discarding the supernatant, the cell pellet with lysis buffer was vortexed. After centrifugation at 13,000 rpm for 15 minutes at 4 °C, the proteasome activity was assayed according to the manufacturer’s protocol (20S Proteasome Activity Assay Kit, Millipore). Proteasome activity values were measured in a fluorometer; assays were performed in triplicate.

### Immunofluorescence staining

To study whether ARV p17 promotes formation of autophagosomes, GFP-LC3 plasmid was used to observe LC3 punta under a fluorescence microscope. This plasmid was provided by Dr. Shieh at the Institute of Biomedical Sciences, National Chung Hsing University, Taiwan. Vero cells were seeded on cover glasses one day before treatment. In the positive controls, Vero cells were pretreated with TG (3 nM) followed by transfection with the GFP-LC3 plasmid for 18 hours. In the case of ARV-infected cells, cells were transfected with GFP-LC3 plasmid for 18 hours followed by infection with ARV at an MOI of 10 for 6 hours. In the case of cells transfected with plasmids, cells were transfected with GFP-LC3 plasmid for 6 hours followed by transfection with plasmids for 18 hours. All treated- and untreated-cells were fixed by 4% paraformaldehyde and nuclei stained with 0.01% DAPI for 10 min. After washing twice with PBS, the cells were observed under the fluorescence microscope.

### Electrophoresis and Western blot assays

Cells were seeded in 6-well cell culture dishes one day before treating with drug or infected/transfected with virus/plasmid as described above. Collected cells were washed with 1X PBS and lysed with lysis buffer. After centrifugation of lysates at 13,000 rpm for 15 minutes at 4 °C, the supernatant was transferred to a new Eppendorf tube for determination of the concentration of solubilized protein with the Bio-Rad Protein Assay (Bio-Rad Laboratories, USA). Assays were performed according to the manufacturer’s protocol. Equal amounts of samples were mixed with 2.5X Lammeli loading buffer and boiled for 10 minutes in a water bath. The proteins were separated in a 10% sodium dodecyl sulphate (SDS)-polyacrylamide gel electrophoresis (PAGE) gel and transferred to a PVDF membrane (GE Healthcare Life Sciences). Expression of individual proteins was determined using the corresponding primary antibody and visualization by horseradish peroxidase (HRP) conjugated secondary antibody. The results were detected on film (GE Healthcare Life Sciences) after membrane incubation with enhanced chemiluminescence reagent (ECL plus) (Amersham Biosciences, UK). The intensity of target proteins was calculated using Photocapt (Vilber Lourmat, France).

### Virus titration

To determine the effects of PSMB6, CDK2, and Akt on ARV replication, shRNAs were used to knockdown the targets in ARV-infected Vero cells. In the case of the PSMB6 knockdown, individual 24-well plates of Vero cells were transfected with a PSMB6 shRNA for 6 hours, followed by ARV infection at an MOI of 5 for 24 hours. In the case of Akt and CDK2 knockdown as well as CDK2 overexpression, individual 24-well plates of Vero cells were infected with ARV at an MOI of 5 for 6 hours, followed by transfection with Akt and CDK2 shRNA or the pCI-neo-CDK2 plasmid for 24 hours, respectively. ARV-infected cell supernatants were collected at 24 hpi. Virus titers were determined by an agar overlay plaque assay performed in triplicate as described^23^.

### Statistical analysis

All data from co-immunoprecipitation assay and other assays were evaluated for statistical significance using the Student’s *t*-test. Data are expressed as mean ± SD of at least three independent experiments^23, 24^. In all tests, p < 0.05 was considered statistically significant.

## Electronic supplementary material

Supplementary Information
