# Supplementary material for: Avian reovirus p17 and σA act cooperatively to downregulate Akt by suppressing mTORC2 and CDK2/cyclin A2 and upregulating proteasome PSMB6
Source: Sci Rep. 2017 Jul 12;7:5226. doi: 10.1038/s41598-017-05510-x (PMC5507987; doi:10.1038/s41598-017-05510-x)

Supplementary Information

Avian reovirus p17 and σA act cooperatively to downregulate Akt by suppressing mTORC2 and CDK2/cyclinA2 and upregulating proteasome PSMB6

Wei-Ru Huang[1], Pei-I Chi[1], Hung-Chuan Chiu, Jue-Liang Hsu, Brent L. Nielsen, Tsai-Ling Liao, Hung-Jen Liu*

*Corresponding author. Phone: 886-4-22840485 ext. 243; Fax: 886-4-22874879; e-mail address: hjliu5257@nchu.edu.tw.

[1] Equal contribution to this work.

Table S1. Expression levels of ribosome and proteasome proteins in ARV-infected Vero cells and proteins identified on 2-DE gels and sequenced through MALDI-TOF mass

Protein name		Protein name	
**60S ribosomal protein**		**40S ribosomal protein**	
Ribosomal protein L3	--	Ribosomal protein S2	-
Ribosomal protein L4	-	Ribosomal protein S3	a
Ribosomal protein L5	-	Ribosomal protein S3a	+++
Ribosomal protein L7	---	Ribosomal protein S4	a
Ribosomal protein L7a	-	Ribosomal protein S5	a
Ribosomal protein L9	-	Ribosomal protein S6	-
Ribosomal protein L10	---	Ribosomal protein S7	-
Ribosomal protein L10a	a	Ribosomal protein S8	-
Ribosomal protein L11	--	Ribosomal protein S9	a
Ribosomal protein L12	-	Ribosomal protein S11	a
Ribosomal protein L13	--	Ribosomal protein S13	+
Ribosomal protein L14	a	Ribosomal protein S14	a
Ribosomal protein L15	a	Ribosomal protein S15	-
Ribosomal protein L18	+	Ribosomal protein S15a	--
Ribosomal protein L18a	a	Ribosomal protein S16	-
Ribosomal protein L19	---	Ribosomal protein S17	a
Ribosomal protein L21	-	Ribosomal protein S18	+
Ribosomal protein L22	a	Ribosomal protein S19	a
Ribosomal protein L23	-	Ribosomal protein S20	-
Ribosomal protein L23a	-	Ribosomal protein S21	+
Ribosomal protein L24	++	Ribosomal protein S24	--
Ribosomal protein L26	---	Ribosomal protein S26	--
Ribosomal protein L27	---	Ribosomal protein S27a	+
Ribosomal protein L28	--	Ribosomal protein S28	a
Ribosomal protein L30	+	Ribosomal protein SA	a
Ribosomal protein L31	-		
Ribosomal protein L31a	--	**Ribosome associated protein**	
Ribosomal protein L34	-	Ribosome binding protein 1	---
Ribosomal protein L35	--	Ribosome maturation protein SBDS	-
Ribosomal protein L35a	-		
Ribosomal protein L36	a		
Ribosomal protein L37a	+++		

Table S1 *(continued)*

Protein name		Protein name	
**19S Proteasome**		**20S Proteasome**	
26S proteasome regulatory subunit 4	a	Proteasome subunit alpha type-1	a
26S proteasome regulatory subunit 6B	-	Proteasome subunit alpha type-2	a
26S proteasome regulatory subunit 7	a	Proteasome subunit alpha type-3	a
26S proteasome regulatory subunit 8	a	Proteasome subunit alpha type-4	+
26S proteasome regulatory subunit 10	a	Proteasome subunit alpha type-5	+
26S proteasome non-ATPase regulatory subunit 1	-	Proteasome subunit alpha type-7	a
26S proteasome non-ATPase regulatory subunit 2	a	Proteasome subunit beta type-1	a
26S proteasome non-ATPase regulatory subunit 3	-	Proteasome subunit beta type-2	a
26S proteasome non-ATPase regulatory subunit 6	a	Proteasome subunit beta type-3	+
26S proteasome non-ATPase regulatory subunit 6A	-	Proteasome subunit beta type-4	+
26S proteasome non-ATPase regulatory subunit 7	a	Proteasome subunit beta type-5	++
26S proteasome non-ATPase regulatory subunit 11	-	Proteasome subunit beta type-6	+++
26S proteasome non-ATPase regulatory subunit 13	a	Proteasome subunit beta type-7	+
26S proteasome non-ATPase regulatory subunit 14	a		

a, No significant change; +, increase < 1.5 fold; ++, increase >1.5 to 2 fold; +++, increase > 2 fold; -, Decrease <1.5 fold; --, decrease > 1.5 to 2 fold; ---, decrease > 2 fold.

Table S2. Primers for real-time PCR for amplification of proteasomal subunits

Gene	Accession number	Sequence (5′-3′)*	Location	Expected size (bp)
PSMB5	NM_001260845	F : GGCCACCTTCTCTGTAGGTTCTGG<br>R : GCCATCCTCCCGCACGTGGTAGAG	564-587<br>744-721	181
PSMB6	NM_001195714	F : TACCTACCGGGAAGGCATGA<br>R : TCCCAAAAGTACTTGCCGCT	543-562<br>693-674	151
PSMB7	NM_001265663	F : ACACAGACATGACAACCCAGCTCATTT<br>R : TAGATGCTGTACAGGTGAGGTCCAGTA	301-327<br>646-623	346
PSMB8	XM_003897422	F: CTCTCTATGGGCAGCATGATCTGTG<br>R: GGTCATAGGCCTCTTCAGGGCTAAG	447-471<br>627-630	184
PSMB9	NM_001194864	F: GCTGGCTGGGACCAACATGAAGG<br>R: GAGCAATAGCGTCTGTGGTGAAG	394-416<br>552-574	181
PSMB10	NM_001194028	F: GGCGGTACTAGAAGACCGGTTCCAG<br>R: GGGCTCTGTGGGTGAGCTCAGTGTC	629-653<br>785-809	181

F: forward primers ; R:reverse primers

Supplementary figure legends

Fig. S1. p17 promotes MDM2 phosphorylation at S166. (A) Agarose gel electrophoresis of semi-quantitative RT-PCR products. To examine whether the MDM2 and Rpl26 transcription was down-regulated by p17, MDM2 and Rpl Rpl26 mRNA levels in ARV-infected and pcDNA3.1-p17-transfected Vero cells were compared with those in uninfected cells alone. (B) The level of p-MDM2 (S166) was examined in Akt-depleted cells. The protein levels were normalized to those for β-actin. The levels of indicated proteins at mock were considered 1-fold. The activation and inactivation folds indicated below each lane were normalized against those at mock. The uncropped blots with molecular weights are shown in Fig. S10.

Fig. S2. ARVσA protein upregulates PSMB6. Vero cells were either infected with ARV at an MOI of 5 or transfected with pcDNA3.1-flag-p17 or pcDNA3.1-flag-σA plasmids for 24 hours followed by Western blot assay with the indicated antibodies. β-actin was included as a loading control. The level of indicated proteins in the mock control was considered 1 fold. The fold activation and inactivation indicated below each lane were normalized against values for the mock control. The uncropped blots with molecular weights are shown in Fig. S10.

Fig. S3. p17 reduces the phosphorylated form of rictor. Both Vero and DF-1 cells were transfected with pcDNA3.1-flag-p17 plasmid for 24 hours followed by Western blot assay with indicated antibodies. In the negative controls, cells were transfected with pcDNA3.1-flag vector for 24 hours. Phosphorylation and protein levels were determined by immunoblotting with the indicated antibodies. The protein levels were normalized to those for β-actin. The level of the indicated proteins at 0h in each panel

was considered 1-fold. The activation and inactivation folds indicated below each lane were normalized against values for the mock control (0h). The uncropped blots with molecular weights are shown in Fig. S10.

Fig. S4. Downregulation of CDK2 in p17-transfected cells. (A) The levels of CDK2, cyclin A, p-Akt (S473), p-GSK3α (S21), p-GSK3β (S9), and p-Rb(S249) in ARV-infected and pcDNA3.1-flag-p17-transfected DF-1 cells were examined by Western blot analysis. pcDNA3.1-flag-p17(1-118)-transfected and mock-infected groups were used as negative controls. β-actin was included as a loading control. (B) Expression and purification of TrxA-His-p17, TrxA-His-17(1-118), GST-CDK2, and GST-cyclin A2. Purified proteins were electrophoresed in 12% polyacrylamide gels. (C) To explore whether ARV p17 binds and inhibits CDK2 kinase activity and Rb phosphorylation at Ser249, an *in vitro* kinase assay using Rb as a substrate was performed. TrxA-His-17(1-118) mutant and BSA were used as negative controls. (D) The levels of p-Akt (S473) in CDK2-depleted cells were examined. The CDK2 shRNA plasmid-transfected cells were collected at 24 hours post transfection, and whole cell lysates were harvested for Western blot assays. β-actin was included as a loading control. The level of indicated proteins in the mock control or at 0h was considered 1 fold. The fold activation and inactivation indicated below each lane were normalized against values for the mock control. The uncropped gels and blots with molecular weights are shown in Fig. S10.

Fig. S5. Original images of blots with molecular weights (KDa).

Fig. S6. Original images of blots with molecular weights (KDa).

Fig. S7. Original images of blots with molecular weights (KDa).

Fig. S8. Original images of blots with molecular weights (KDa).

Fig. S9. Original images of blots with molecular weights (KDa).

Fig. S10. Original images of gels and blots with molecular weights (KDa).

Figure S1

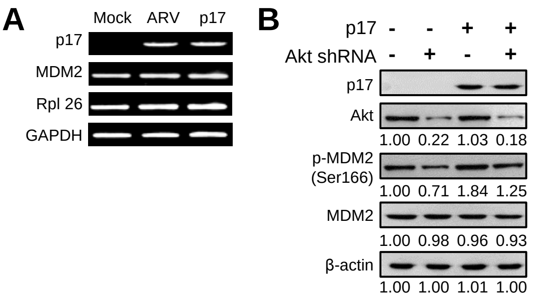
A
Mock
ARV
p17
p17
MDM2
Rpl 26
GAPDH
B
p17 - - + +
Akt shRNA - + - +
p17
Akt
1.00 0.22 1.03 0.18
p-MDM2
(Ser166)
1.00 0.71 1.84 1.25
MDM2
1.00 0.98 0.96 0.93
β-actin
1.00 1.00 1.01 1.00

Figure S2

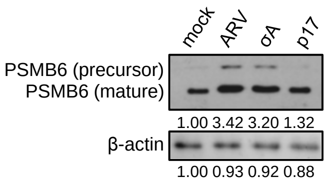

mock
ARV
σA
p17
PSMB6 (precursor)
PSMB6 (mature)
1.00 3.42 3.20 1.32
β-actin
1.00 0.93 0.92 0.88

Figure S3

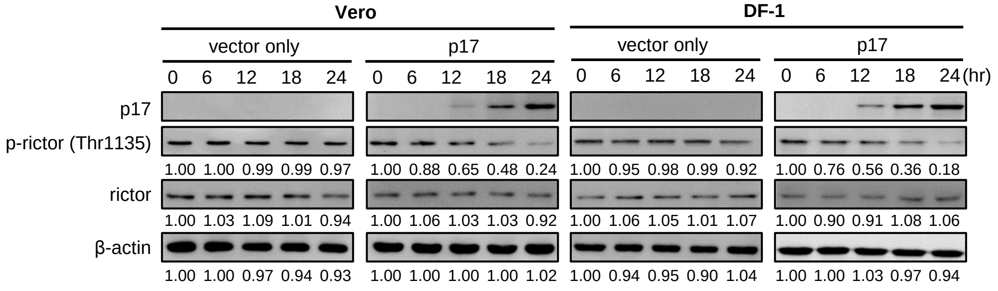
Vero
DF-1
vector only
p17
vector only
p17
0 6 12 18 24 0 6 12 18 24 0 6 12 18 24 0 6 12 18 24 (hr)
p17
p-rictor (Thr1135)
1.00 1.00 0.99 0.99 0.97 1.00 0.88 0.65 0.48 0.24 1.00 0.95 0.98 0.99 0.92 1.00 0.76 0.56 0.36 0.18
rictor
1.00 1.03 1.09 1.01 0.94 1.00 1.06 1.03 1.03 0.92 1.00 1.06 1.05 1.01 1.07 1.00 0.90 0.91 1.08 1.06
β-actin
1.00 1.00 0.97 0.94 0.93 1.00 1.00 1.00 1.00 1.02 1.00 0.94 0.95 0.90 1.04 1.00 1.00 1.03 0.97 0.94

Figure S4

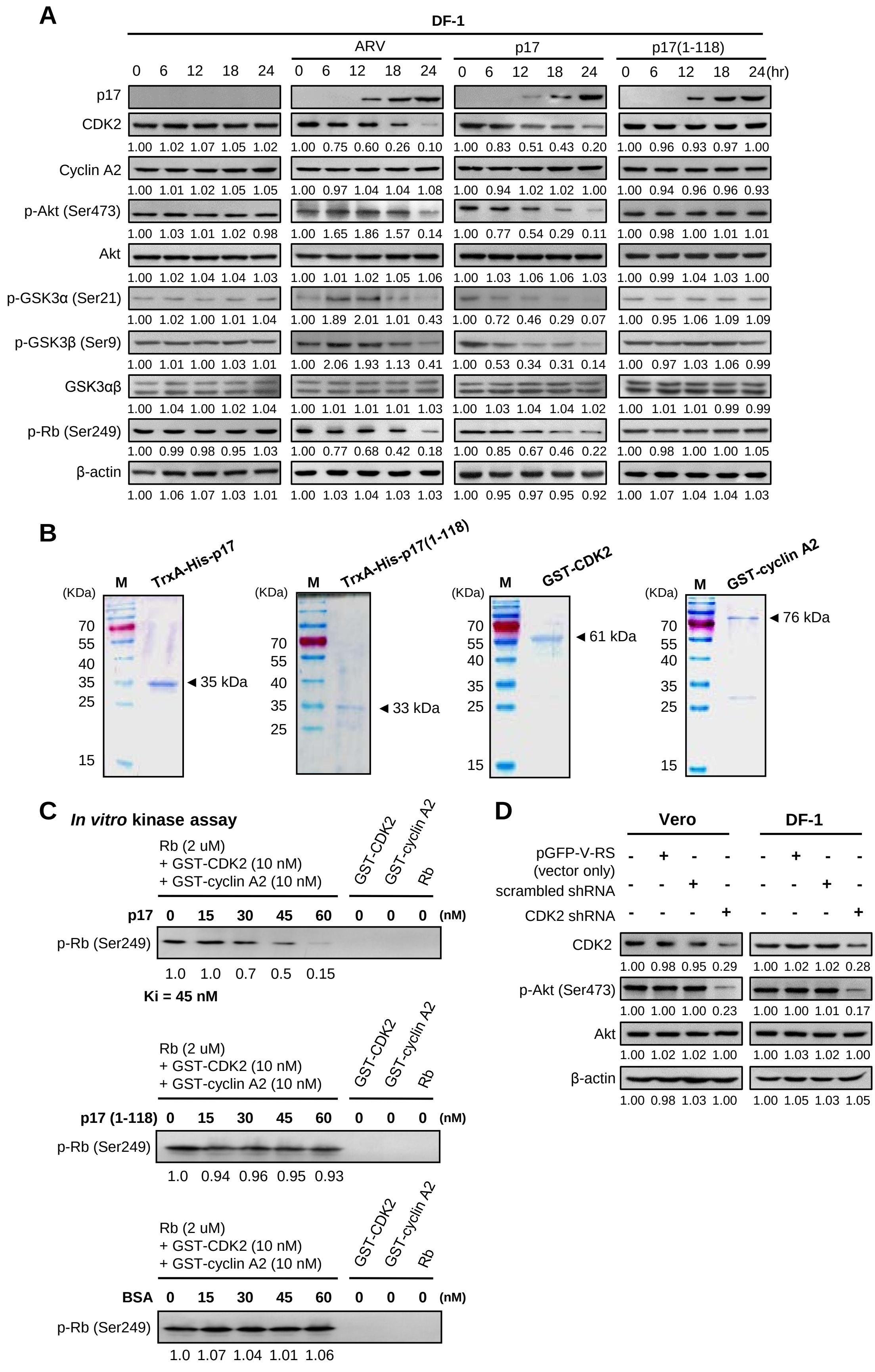
A
DF-1
ARV
p17
p17(1-118)
0 6 12 18 24 0 6 12 18 24 0 6 12 18 24 0 6 12 18 24(hr)
p17
CDK2
1.00 1.02 1.07 1.05 1.02 1.00 0.75 0.60 0.26 0.10 1.00 0.83 0.51 0.43 0.20 1.00 0.96 0.93 0.97 1.00
Cyclin A2
1.00 1.01 1.02 1.05 1.05 1.00 0.97 1.04 1.04 1.08 1.00 0.94 1.02 1.02 1.00 1.00 0.94 0.96 0.96 0.93
p-Akt (Ser473)
1.00 1.03 1.01 1.02 0.98 1.00 1.65 1.86 1.57 0.14 1.00 0.77 0.54 0.29 0.11 1.00 0.98 1.00 1.01 1.01
Akt
1.00 1.02 1.04 1.04 1.03 1.00 1.01 1.02 1.05 1.06 1.00 1.03 1.06 1.06 1.03 1.00 0.99 1.04 1.03 1.00
p-GSK3α (Ser21)
1.00 1.02 1.00 1.01 1.04 1.00 1.89 2.01 1.01 0.43 1.00 0.72 0.46 0.29 0.07 1.00 0.95 1.06 1.09 1.09
p-GSK3β (Ser9)
1.00 1.01 1.00 1.03 1.01 1.00 2.06 1.93 1.13 0.41 1.00 0.53 0.34 0.31 0.14 1.00 0.97 1.03 1.06 0.99
GSK3αβ
1.00 1.04 1.00 1.02 1.04 1.00 1.01 1.01 1.01 1.03 1.00 1.03 1.04 1.04 1.02 1.00 1.01 1.01 0.99 0.99
p-Rb (Ser249)
1.00 0.99 0.98 0.95 1.03 1.00 0.77 0.68 0.42 0.18 1.00 0.85 0.67 0.46 0.22 1.00 0.98 1.00 1.00 1.05
β-actin
1.00 1.06 1.07 1.03 1.01 1.00 1.03 1.04 1.03 1.03 1.00 0.95 0.97 0.95 0.92 1.00 1.07 1.04 1.04 1.03
B
(KDa) M TrxA-His-p17
70 55 40 35 25 15
◄ 35 kDa
(KDa) M TrxA-His-p17(1-118)
70 55 40 35 25
◄ 33 kDa
(KDa) M GST-CDK2
70 55 40 35 25 15
◄ 61 kDa
(KDa) M GST-cyclin A2
70 55 40 35 25 15
◄ 76 kDa
C In vitro kinase assay
Rb (2 uM)
+ GST-CDK2 (10 nM)
+ GST-cyclin A2 (10 nM)
GST-CDK2 GST-cyclin A2 Rb
p17 0 15 30 45 60 0 0 0 (nM)
p-Rb (Ser249)
1.0 1.0 0.7 0.5 0.15
Ki = 45 nM
Rb (2 uM)
+ GST-CDK2 (10 nM)
+ GST-cyclin A2 (10 nM)
GST-CDK2 GST-cyclin A2 Rb
p17 (1-118) 0 15 30 45 60 0 0 0 (nM)
p-Rb (Ser249)
1.0 0.94 0.96 0.95 0.93
Rb (2 uM)
+ GST-CDK2 (10 nM)
+ GST-cyclin A2 (10 nM)
GST-CDK2 GST-cyclin A2 Rb
BSA 0 15 30 45 60 0 0 0 (nM)
p-Rb (Ser249)
1.0 1.07 1.04 1.01 1.06
D
Vero
DF-1
pGFP-V-RS (vector only) - + - - - + - -
scrambled shRNA - - + - - - + -
CDK2 shRNA - - - + - - - +
CDK2
1.00 0.98 0.95 0.29 1.00 1.02 1.02 0.28
p-Akt (Ser473)
1.00 1.00 1.00 0.23 1.00 1.00 1.01 0.17
Akt
1.00 1.02 1.02 1.00 1.00 1.03 1.02 1.00
β-actin
1.00 0.98 1.03 1.00 1.00 1.05 1.03 1.05

Figure S5

Fig. 1A

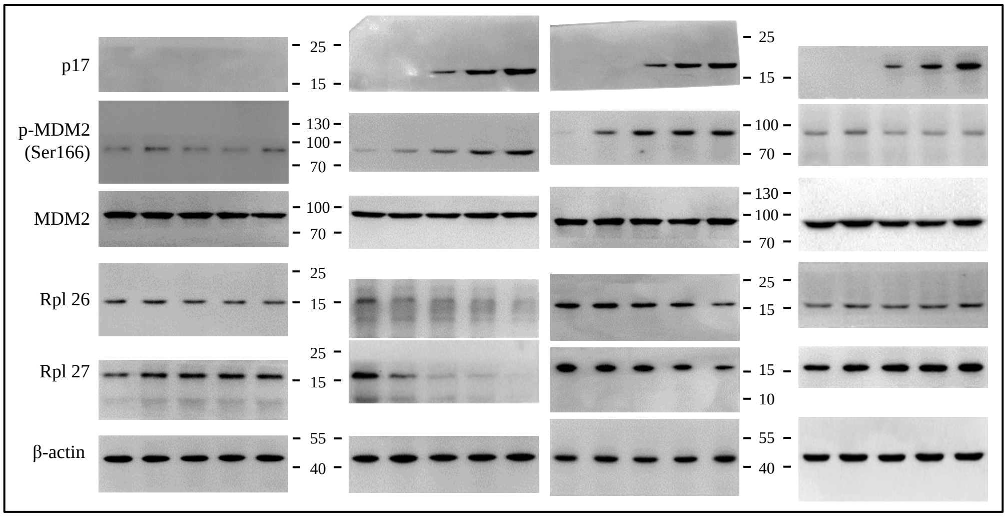

Fig. 1B

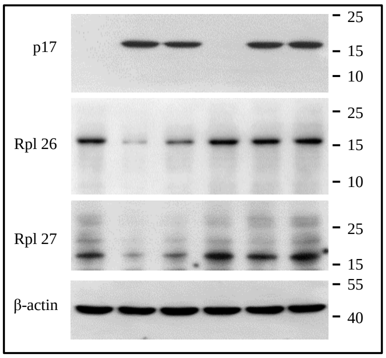

Fig. 1C

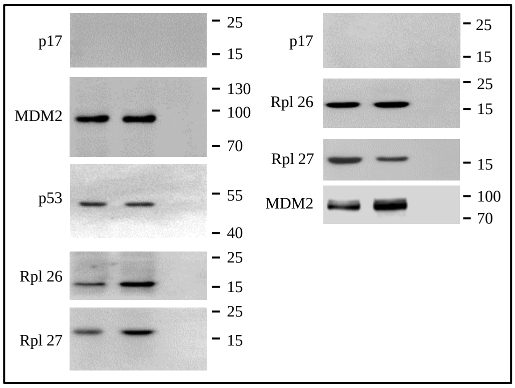

Fig. 1D

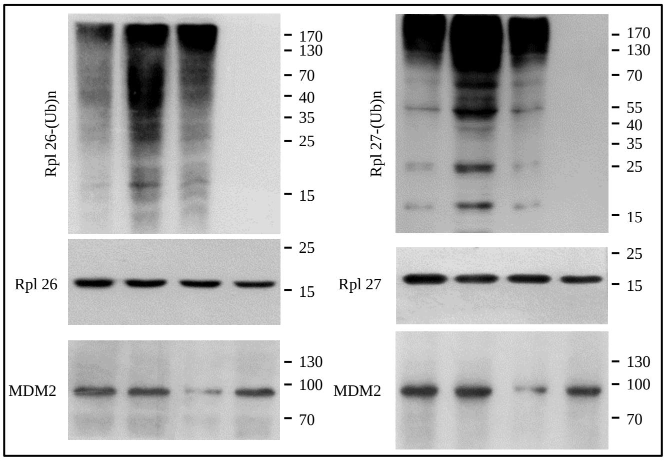

Fig. 1E

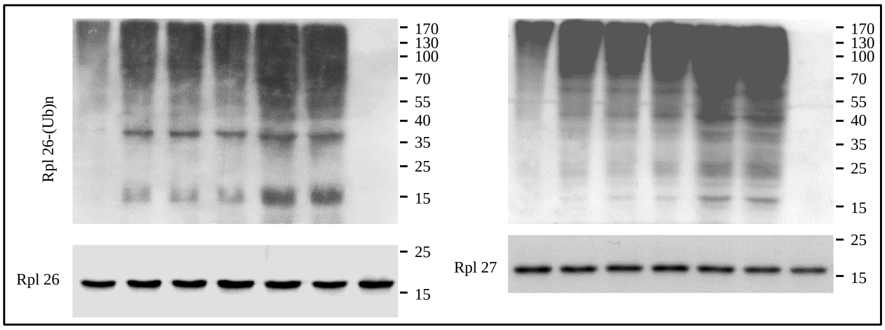

Fig. 2E

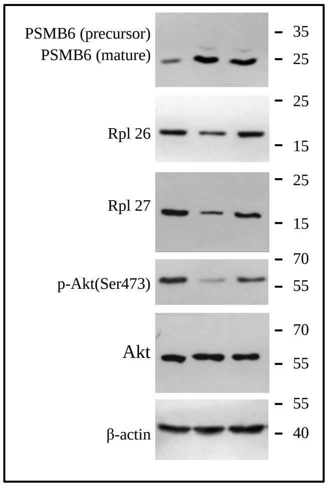

Fig. 2B

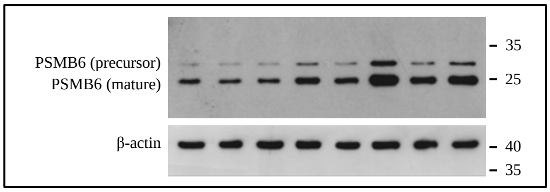

Fig. 2D

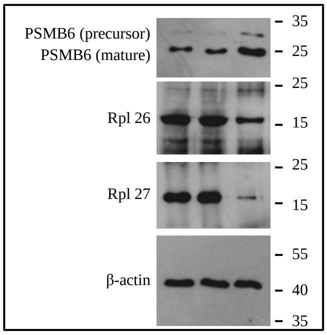

Figure S6

Fig. 2F

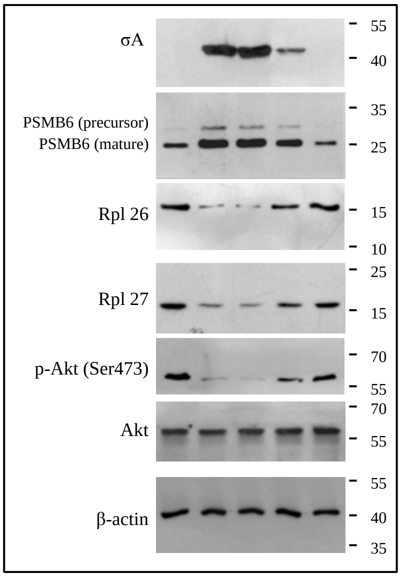

Fig. 2G

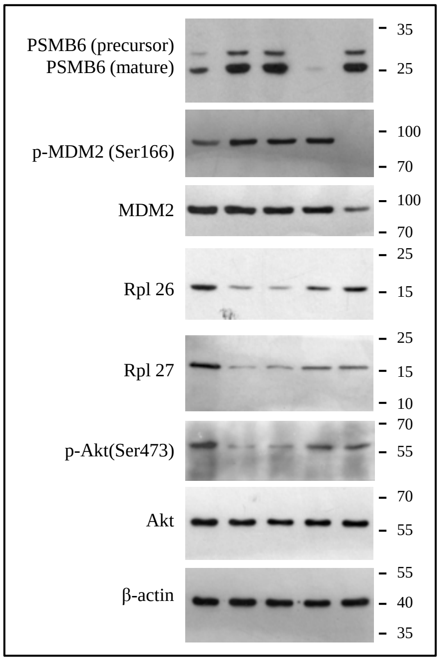

Fig. 2H

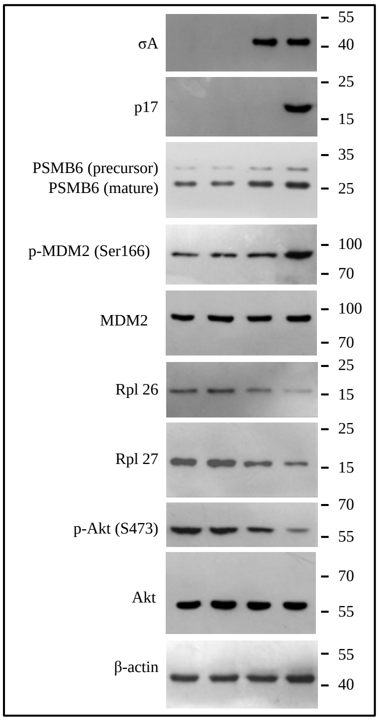

Fig. 3A

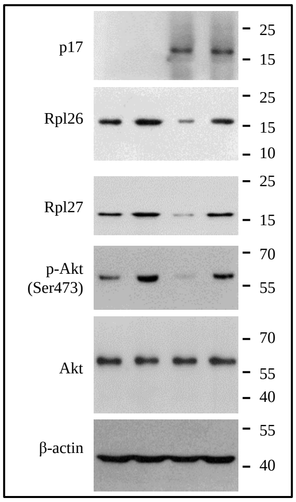

Fig. 3B

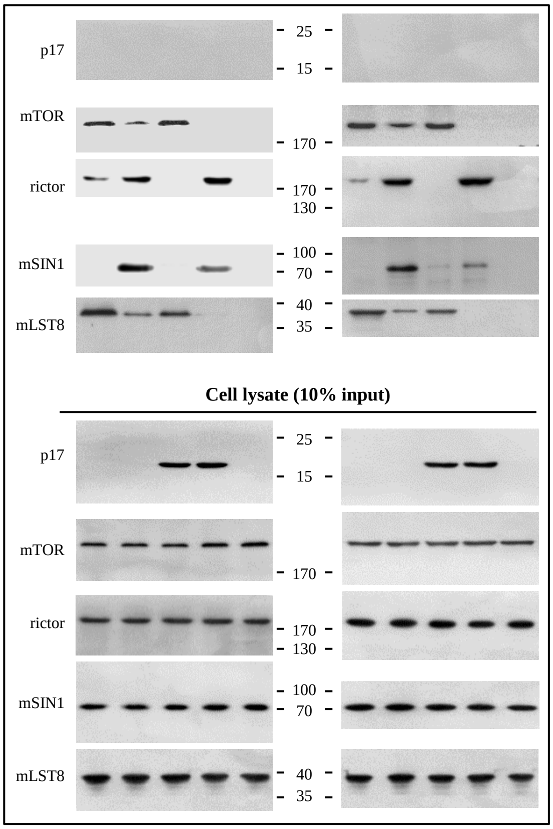

Figure S7

Fig. 3C

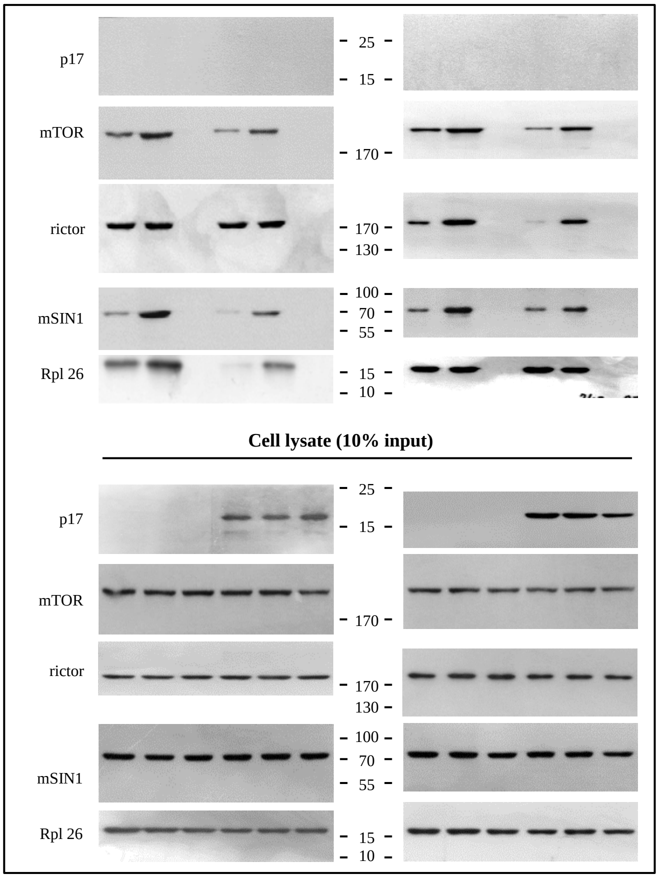

Fig. 3D

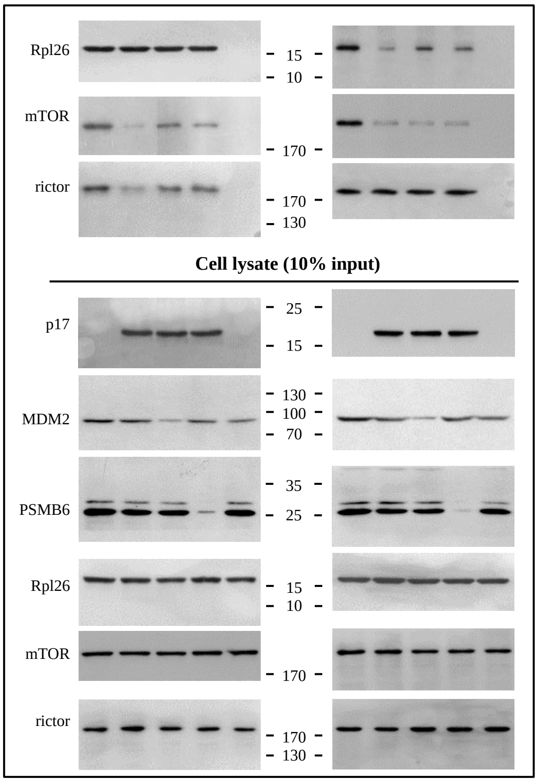

Fig. 4A

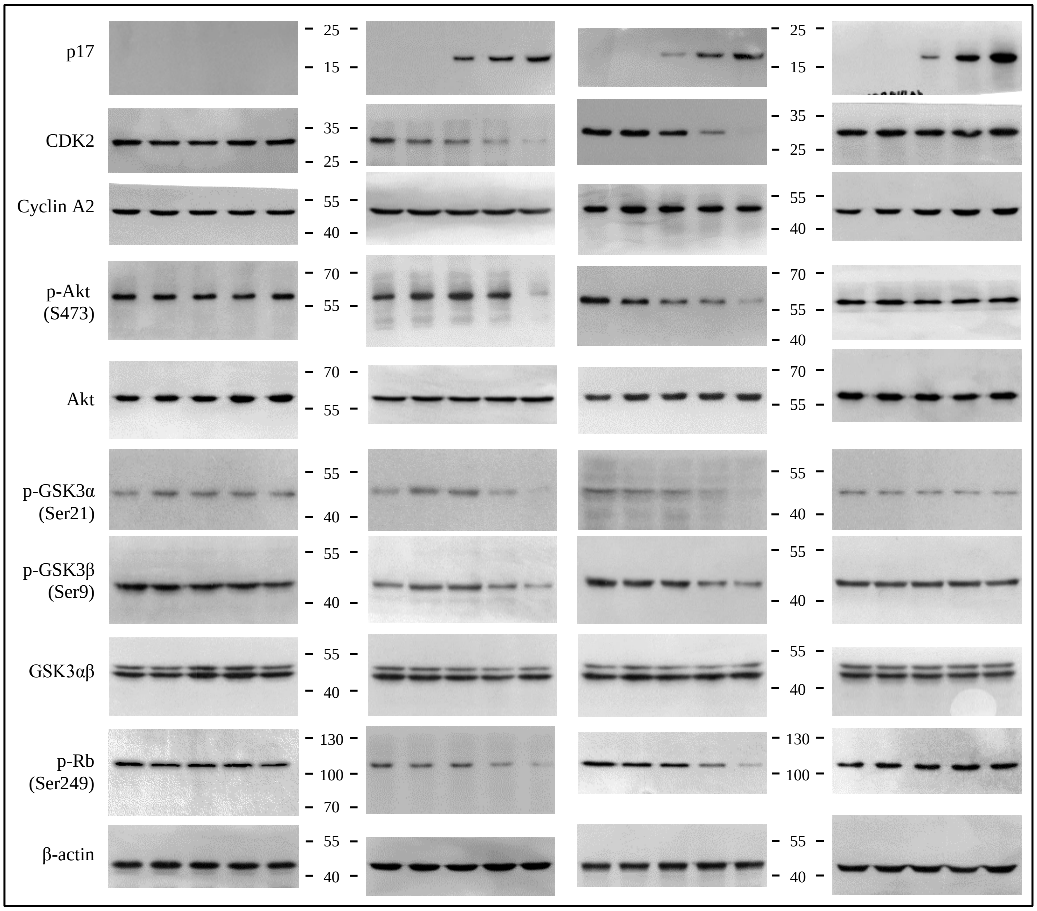

Figure S8

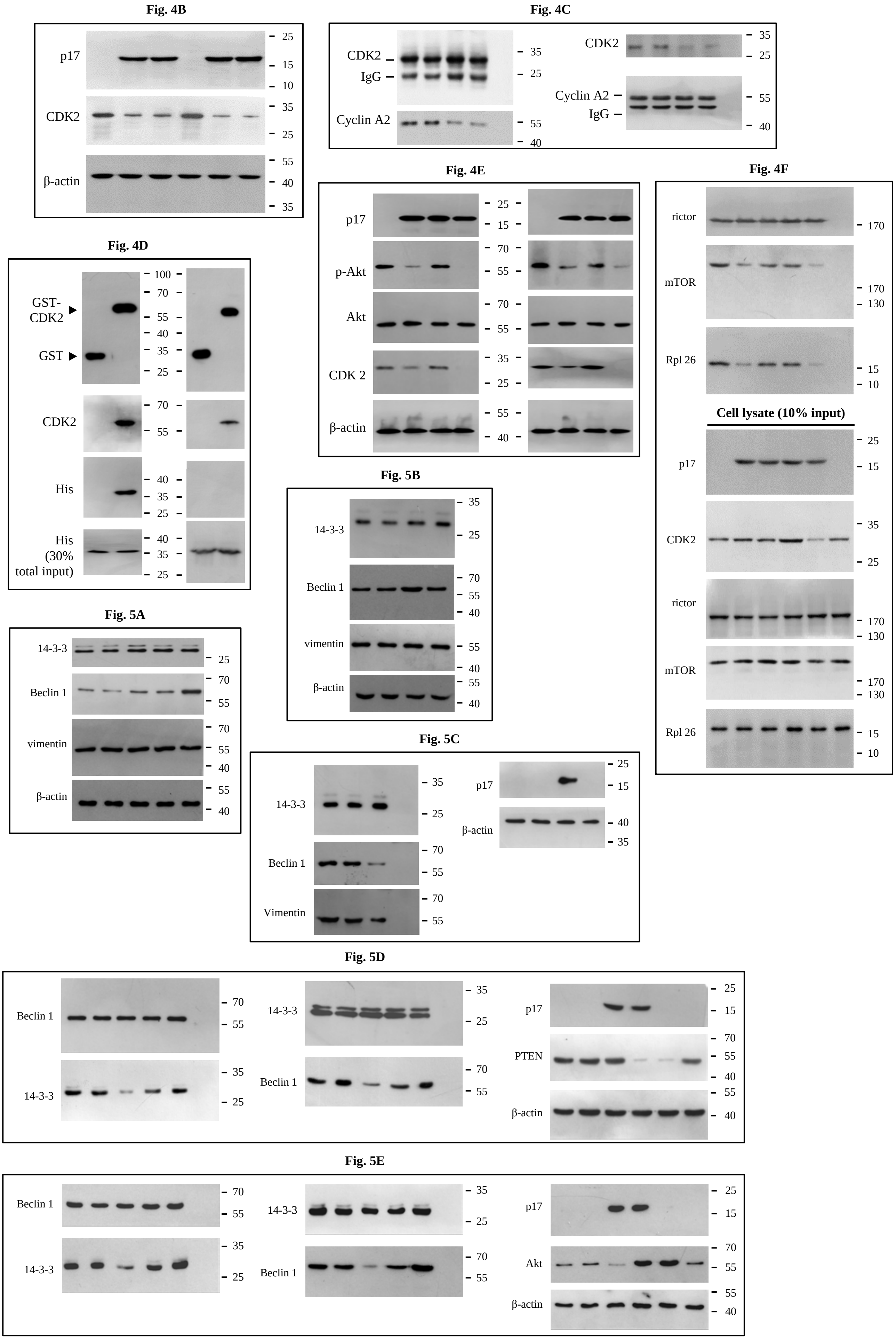

Fig. 4B
p17
CDK2
β-actin
Fig. 4C
CDK2
IgG
Cyclin A2
CDK2
Cyclin A2
IgG
Fig. 4D
GST-CDK2
GST
CDK2
His
His (30% total input)
Fig. 4E
p17
p-Akt
Akt
CDK 2
β-actin
Fig. 4F
rictor
mTOR
Rpl 26
Cell lysate (10% input)
p17
CDK2
rictor
mTOR
Rpl 26
Fig. 5A
14-3-3
Beclin 1
vimentin
β-actin
Fig. 5B
14-3-3
Beclin 1
vimentin
β-actin
Fig. 5C
14-3-3
Beclin 1
Vimentin
p17
β-actin
Fig. 5D
Beclin 1
14-3-3
14-3-3
Beclin 1
p17
PTEN
β-actin
Fig. 5E
Beclin 1
14-3-3
14-3-3
Beclin 1
p17
Akt
β-actin

Figure S9

Fig. 5F

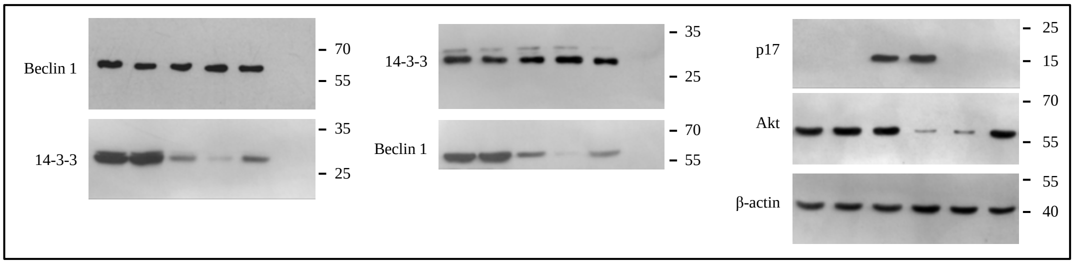

Fig. 5G

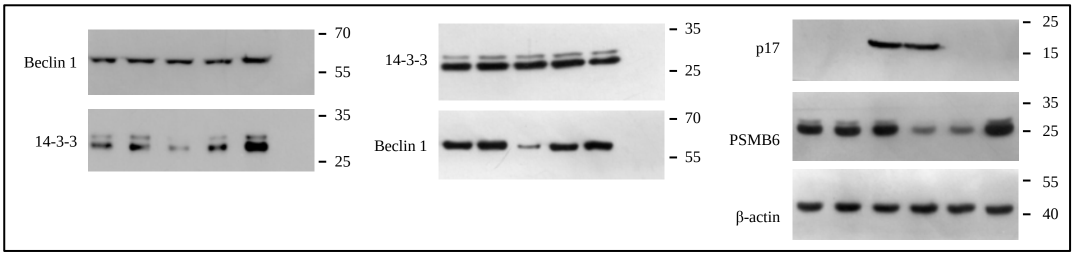

Fig. 5H

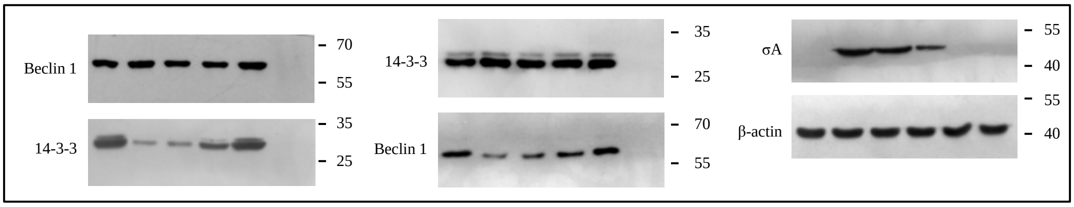

Fig. 6C

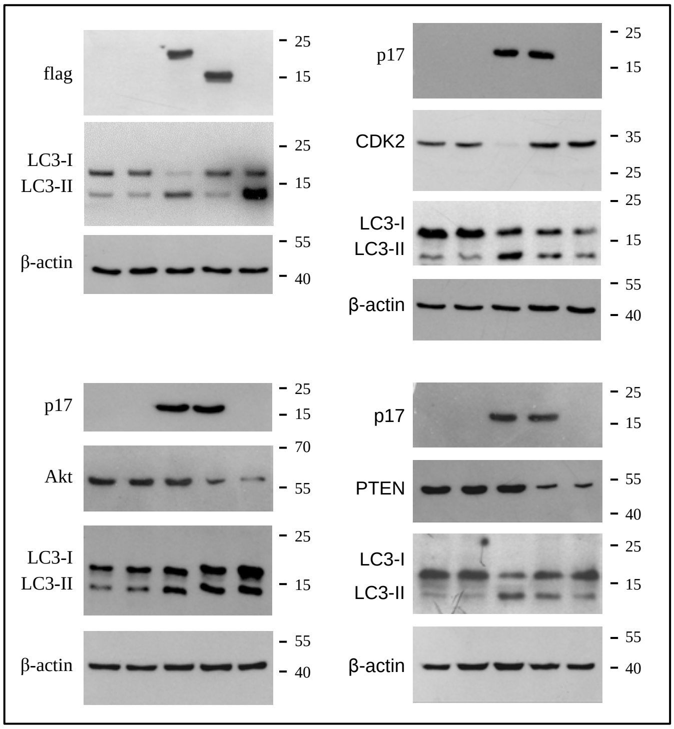

Fig. S1B

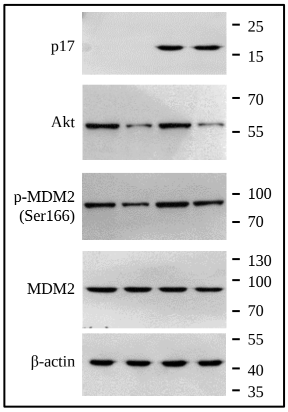

Fig. S2

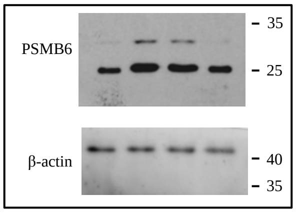

Fig. S3

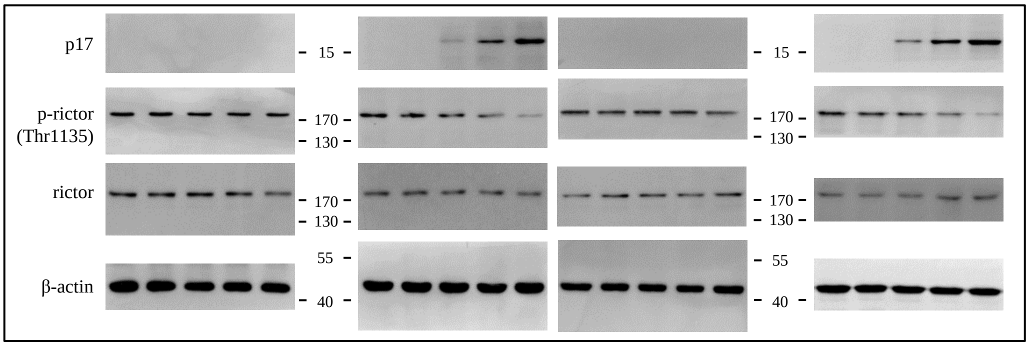

Figure S10

Fig. S4A

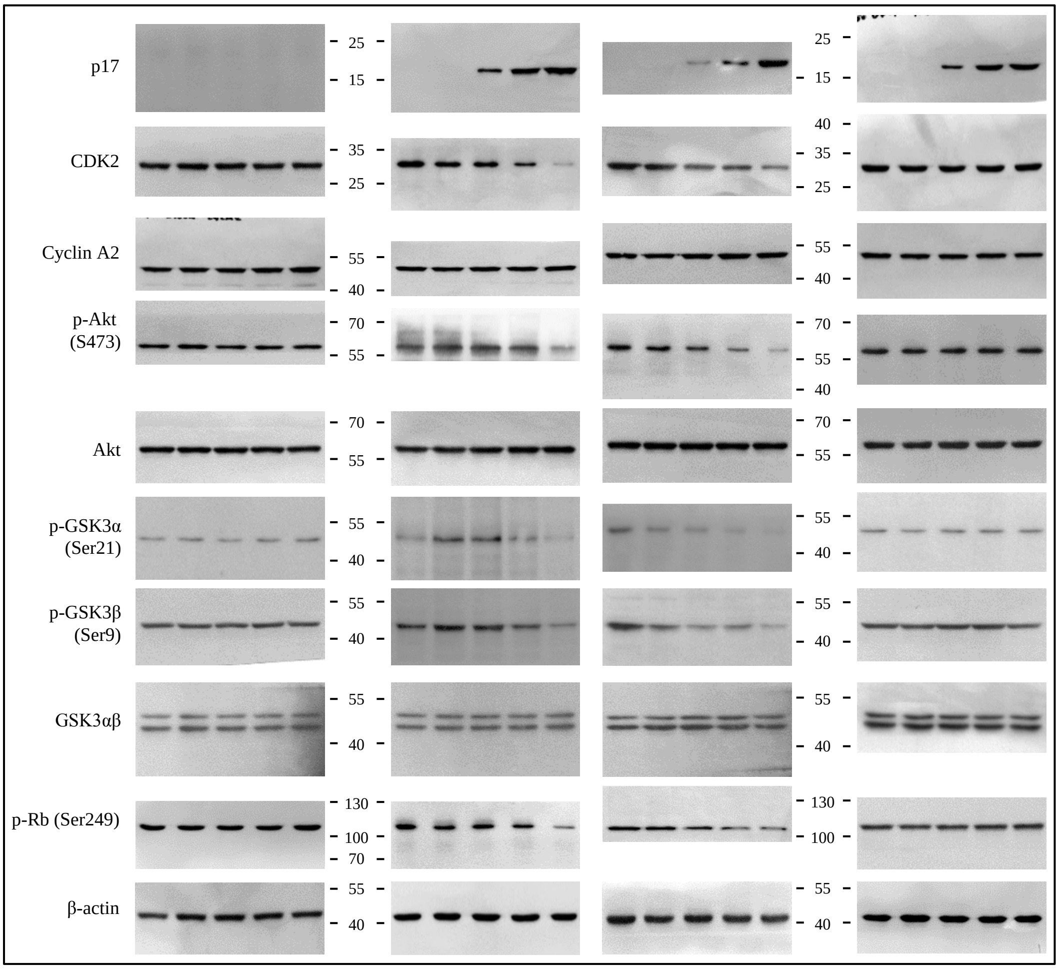

Fig. S4B

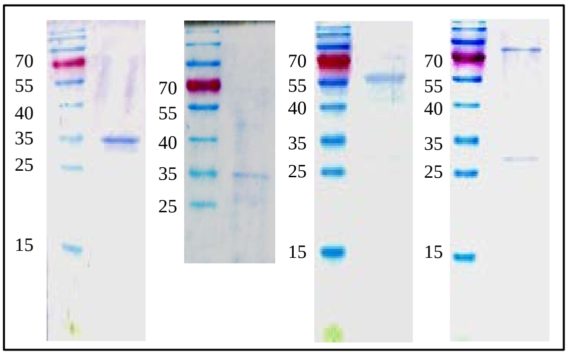

Fig. S4C

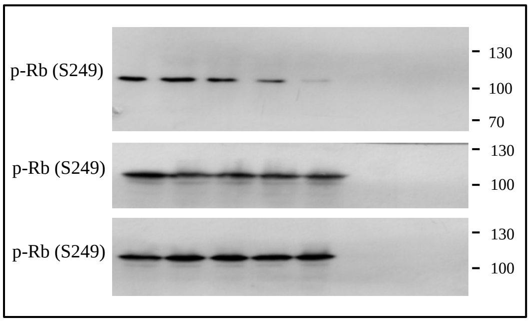

Fig. S4D

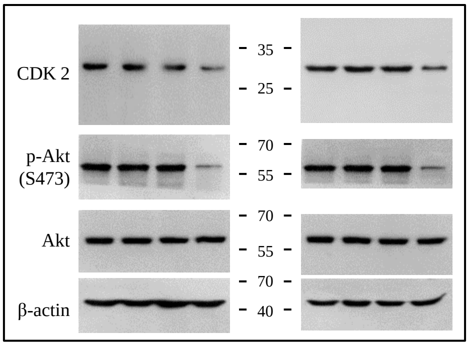

Supplement: Supplementary file 1 — Supplementary Information [file 41598_2017_5510_MOESM1_ESM.pdf]
